# Alicyclobacillin 24: a class III bacteriocin from *Alicyclobacillus acidoterrestris* targeting species associated with spoilage of acidic fruit-based products

**DOI:** 10.3389/fmicb.2026.1823210

**Published:** 2026-05-01

**Authors:** Inês Carvalho Leonardo, Ana Patrícia Quendera, Micael C. Freitas, Sandra Pereira Santos, Ana R. Lemos, Carolina Ferro Rodrigues, Helena Ferreira, Margarida Sobral Pimenta, Carlos São-José, Tiago M. Bandeiras, Maria Teresa Barreto Crespo, Frédéric Bustos Gaspar

**Affiliations:** 1 Instituto de Biologia Experimental e Tecnológica (iBET), Oeiras, Portugal; 2 Instituto de Tecnologia Química e Biológica António Xavier, Universidade Nova de Lisboa, Oeiras, Portugal; 3 Research Institute for Medicines (iMed.ULisboa), Faculdade de Farmácia, Universidade de Lisboa, Lisboa, Portugal

**Keywords:** antibacterial activity, bacteriocin, biopreservation, fruit juice spoilage, thermophilic bacteria

## Abstract

The thermoacidophilic *Alicyclobacillus* (ACB) genus includes several species responsible for spoilage of acidic fruit-based products, yet bacteriocins produced by these bacteria remain unexplored. In this study, the spoilage strain *Alicyclobacillus acidoterrestris* DSM 3922^T^ was explored as a potential bacteriocin producer, following genome analysis that revealed the presence of a putative geobacillin 26 (Geo26) family protein. Co-culture assays showed inhibitory activity against other ACB isolates, and proteomic analysis confirmed the secretion of a Geo26 homolog, designated alicyclobacillin 24 (Ali24). Ali24 was heterologously expressed with a C-terminal His-tag (Ali24-His), purified, and functionally characterized. Ali24-His exhibited a narrow antibacterial spectrum restricted to ACB species, including multiple spoilage-associated strains. SYTO9/propidium iodide staining of *A. acidoterrestris* MMB007 exposed to Ali24-His suggested a membrane associated antimicrobial mechanism. In addition, the antibacterial activity of Ali24-His was evaluated in industrial-relevant conditions, exhibiting effective inhibitory features under acidic pH and moderate heating (up to 50 °C), which are environments typical of fruit processing workflows, pre-pasteurization steps. Ali24-His stability was further assessed by dynamic light scattering and nano differential scanning fluorimetry revealing enhanced stability and reduced aggregation under acidic conditions, consistent with its activity profile. Overall, this work reports the first functional Class III bacteriocin from *A. acidoterrestris* and expands the repertoire and understanding of thermophile-derived bacteriocins with potential for sustainable control of ACB spoilage in the food industry.

## 1 Introduction

*Alicyclobacillus* (ACB) spp. are Gram-positive, thermoacidophilic bacteria that compromise the quality of fruit-based products by producing medicinal, cheesy, and disinfectant-like off-flavors and odors, ultimately leading to food waste (Gocmen et al., 2005; Leonardo et al., 2025). These bacteria are particularly problematic in the food industry due to their ability to withstand industrial processing conditions, such as acidic environments and pasteurization, typically applied to eliminate pathogenic and spoilage microorganisms. This resilience is further enhanced by the formation of endospores, which are highly resistant to processing steps and can germinate during the product’s shelf-life (Cerny et al., 1984; Deinhard et al., 1987; Sokołowska et al., 2020).

To mitigate ACB-related spoilage events, several strategies have been investigated, including physical, chemical, and biological approaches (Oliveira et al., 2024; Pinto et al., 2024; Cai et al., 2025a; Wang et al., 2025). Among them, bacteriocins have been shown to be a suitable alternative for ACB control, aligning with the current consumer demand for natural preservatives and a more sustainable food production (Pornpukdeewattana et al., 2020; Sourri et al., 2022). Bacteriocins are ribosomally synthesized antimicrobial peptides or proteins produced by bacteria, generally active against closely related species (Meade et al., 2020). They are structurally and functionally diverse and can be broadly classified into four distinct groups (Class I to IV) based on size, structural complexity, and post-translational modifications (Heng and Tagg, 2006; Kumariya et al., 2019; Vaičikauskaitė et al., 2019).

Specifically regarding ACB control, several Class I (small and extensively post-translationally modified peptides) and Class II (small but non-modified peptides) bacteriocins have been tested for their capacity to eliminate both vegetative cells and spores from distinct acidic environments, including laboratory medium and commercial fruit juices of orange, apple, pineapple, peach, and grapefruit (Tianli et al., 2014). Examples include the Class I bacteriocins nisin and bovicin HC5, and the Class II enterocin AS-48, warnericin RB4, bificin C6165, paracin C and plantaricyclin, produced by Gram-positive bacteria, such as *Lactococcus lactis*, *Streptococcus bovis*, *Enterococcus faecalis*, *Staphylococcus warneri*, *Bifidobacterium animalis*, *Lacticaseibacillus paracasei* (formerly *Lactobacillus paracasei*), and *Lactiplantibacillus plantarum* (formerly *Lactobacillus plantarum*), respectively (Grande et al., 2005; Minamikawa et al., 2005; de Carvalho et al., 2008; Ruiz et al., 2013; Huertas et al., 2014; Pei et al., 2014, 2017; Borrero et al., 2017; Sourri et al., 2022). Although promising, most of these studies have focused on the elimination of *Alicyclobacillus acidoterrestris*, the species most frequently reported in spoilage of acidic food products, particularly fruit juices and tomato-derived products, overlooking other ACB species that have also been associated with spoilage (Gocmen et al., 2005; Merle and Montville, 2014; Ciuffreda et al., 2015; Sourri et al., 2022; Leonardo et al., 2025). Moreover, beyond their role as spoilage organisms, ACB bacteria may possess largely unexplored biosynthetic potential for biotechnology useful molecules. Like other thermophilic bacteria, known to produce bacteriocin-like compounds, ACB inhabit extreme environments where antimicrobial compounds production can enhance competitiveness within microbial communities (Mikulík et al., 2017; Kaunietis et al., 2019; Koniuchovaitė et al., 2023; Aireche et al., 2025). Their adaptation to acidic and high temperature environments suggests that ACB species are promising candidates for the production of thermostable antimicrobials targeting similar spoilage microorganisms (Matzke et al., 1997; Satheesh Kumar et al., 2010). However, to date, this possibility remains largely underexplored. Within this framework, the present study investigates whether *A. acidoterrestris* DSM 3922^T^ could itself serve as a source of natural preservatives capable of inhibiting other spoilage-causing ACB strains and species.

Previous genome analysis identified a putative geobacillin 26 (Geo26) family protein in this strain (Leonardo et al., 2022). Geo26, produced by *Geobacillus stearothermophilus*, is a recently characterized heat-labile, high-molecular-weight (MW) antibacterial protein classified as a Class III bacteriocin (Vaičikauskaitė et al., 2019). It exhibits a narrow antibacterial spectrum targeting other thermophilic bacteria, and has been proposed as a natural antibacterial agent with potential for use in the food industry, to be applied in processes where temperatures range from 50 °C to 60 °C (Vaičikauskaitė et al., 2019).

Based on these findings, this study aims to address this knowledge gap by assessing the antimicrobial potential of *A. acidoterrestris* DSM 3922^T^ and characterizing the geobacillin-like protein identified in the genome. Specifically, we (1) evaluate the antibacterial activity of *A. acidoterrestris* DSM 3922^T^ against other ACB species and strains, (2) determine whether this activity correlates with secretion of the predicted bacteriocin, and (3) express, purify, and characterize the recombinant protein with respect to antimicrobial activity and stability under conditions relevant to fruit processing. Together, these objectives aim to expand current knowledge of bacteriocins produced by thermoacidophilic bacteria while exploring new strategies to mitigate ACB spoilage in acidic food systems.

## 2 Materials and methods

### 2.1 Bacterial strains and culture media

The bacterial strains used in this study are listed in Table 1.

**Table 1:** Bacterial strains used in this study.

Bacterial strain	Origin/genotype and phenotype	Reference
Culture collection strains		
*Alicyclobacillus acidiphilus* DSM 14558^T^	Acidic beverage	DSMZ (Germany)
*Alicyclobacillus acidocaldarius* DSM 446^T^	Acid hot spring	DSMZ (Germany)
*Alicyclobacillus acidoterrestris* DSM 3922^T^	Soil	DSMZ (Germany)
*Alicyclobacillus cycloheptanicus* DSM 4006^T^	Soil	DSMZ (Germany)
*Alicyclobacillus fastidiosus* DSM 17978^T^	Apple juice	DSMZ (Germany)
*Bacillus subtilis* CECT 356	Not specified by the collection	CECT (Spain)
*Bacillus cereus* BGSC 6A1	Not specified by the collection	BGSC (United States)
*Escherichia coli* ATCC 25922	Clinical isolate	ATCC (United States)
*Geobacillus stearothermophilus* DSM 22^T^	Deteriorated canned food	DSMZ (Germany)
*Staphylococcus aureus* ATCC 6538	Clinical isolate, human lesion	ATCC (United States)
In-house culture collection strains		
*A. acidocaldarius* MMB020	Tomato paste	This study
*A. acidoterrestris* MMB001	Passion fruit concentrate	This study
*A. acidoterrestris* MMB002	Peach juice	This study
*A. acidoterrestris* MMB003	Peach juice	This study
*A. acidoterrestris* MMB004	Fruit mix concentrate	This study
*A. acidoterrestris* MMB005	Pear juice	This study
*A. acidoterrestris* MMB006	Peach juice	This study
*A. acidoterrestris* MMB007	Tangerine concentrate	This study
*A. acidoterrestris* MMB008	Sugar	This study
*A. acidoterrestris* MMB009	Tangerine concentrate	This study
*A. acidoterrestris* MMB010	Mango and orange juice	This study
*A. acidoterrestris* MMB011	Peach juice	This study
*A. acidoterrestris* MMB012	Peach juice	This study
*A. acidoterrestris* MMB013	Peach juice	This study
*A. acidoterrestris* MMB021	Peach juice	This study
Protein expression strain		
*E. coli* Tuner™ (DE3) pLysS	F^−^ *ompT hsdS*_B_ (r_B_^−^ m_B_^−^) *gal dcm lacY1*(DE3) pLysS (Cam^R^)	Novagen (Germany)

ACB strains, both from international culture collections and from an in-house culture collection comprising isolates recovered from fruit juices and concentrates, tomato-derived products, and other food-processing matrices, were routinely grown in *Bacillus acidoterrestris* (BAT) broth (Scharlau, Germany) supplemented with 1.5% (w/v) agar (VWR, United States) and incubated at 45 °C. Liquid cultures were prepared from fresh colonies grown in BAT broth at the same temperature with shaking at 180 rpm in a New Brunswick Innova® 42R incubator (Germany). Both BAT agar and BAT broth were adjusted to pH 4.0 using a 1 M HCl solution.

*Bacillus subtilis* CECT 356, *Bacillus cereus* BGSC 6A1, *G. stearothermophilus* DSM 22, *Staphylococcus aureus* ATCC 6538, and *Escherichia coli* ATCC 25922 were also included in this study to evaluate the antimicrobial susceptibility of bioactive proteins beyond the ACB genus. They were all grown in Luria-Bertani broth (LB), prepared by adding 10 g/L tryptone (Millipore Sigma, Switzerland), 5 g/L yeast extract (Thermo Scientific, United States), and 10 g/L NaCl (VWR Chemicals, United States), or in Luria-Bertani agar (LA) by supplementing LB with 1.5% (w/v) agar. Incubation was performed at 37 °C with shaking at 180 rpm in a New Brunswick Innova® 42R for liquid cultures, except for *G. stearothermophilus* where incubation was performed at 55 °C.

*E. coli* Tuner™ (DE3) pLysS (Novagen, Germany) was used to express the Geo26 family protein from *A. acidoterrestris* DSM 3922^T^ by cloning the respective gene into a vector pET-21a(+) (Novagen, Germany). This expressing strain was grown in LA and LB supplemented with the appropriate antibiotics at the following concentrations: 25 μg/mL of chloramphenicol (Cam_25_) and 100 μg/mL of ampicillin (Amp_100_). After transformation, this strain was recovered in Super Optimal broth with Catabolite repression (SOC) broth composed of tryptone 20 g/L, yeast extract 5 g/L, 10 mM NaCl, 2.5 mM KCl (Scharlau, Germany), 10 mM MgCl_2_ (VWR, United States), 10 mM MgSO_4_ (Sigma-Aldrich, United States), and 20 mM glucose (Sigma-Aldrich, United States).

### 2.2 Proteome identification by liquid chromatography coupled with mass spectrometry (LC-MS)

A proteomic analysis of the *A. acidoterrestris* DSM 3922^T^ secretome was performed to identify the protein responsible for the observed antibacterial activity. For that, the supernatant of a 24 h culture of *A. acidoterrestris* DSM 3922^T^ was collected after centrifugation at 6,000g for 10 min, then filtered through a 0.45 μm polyethersulfone (PES) syringe filter. ThermoScientific™ Pierce™ protein concentrators with a 9 kDa weight cut-off were used to concentrate the culture supernatant approximately 20-fold. PhosSTOP™ (Roche, Switzerland) and cOmplete™ mini protease inhibitor cocktail (Roche, Switzerland) were added to the concentrated supernatant, following the manufacturer’s instructions. This sample was further analyzed by the UniMS—Mass Spectrometry Unit, ITQB/iBET (Oeiras, Portugal), which provided the MS data. For this, the concentrated supernatant was analyzed by sodium dodecyl sulphate polyacrylamide gel electrophoresis (SDS-PAGE) and gel bands were excised, reduced in 10 mM dithiothreitol (DTT) (Sigma, United States) for 40 min at 56 °C, and alkylated in 55 mM iodoacetamide (Sigma, United States) for 30 min in the dark. Then, an overnight trypsin digestion of proteins was performed at 37 °C according to Correia et al., (2026). The digested and cleaned up sample was analyzed by LC-MS/MS performed on a Waters M-Class HPLC (Waters, United States) coupled to a ZenoTOF 7,600 system (SCIEX, United States) with an OptiFlow Turbo V Ion Source using a micro electrode (1 μL/min to 10 μL/min). Peptides were separated in direct injection mode at 5 μL/min, on a Kinetex XB-C18 analytical column (150 × 0.30 mm, 2.6 μm particle size; Phenomenex, United States). The gradient was as follows: 0.0–2.0 min, 5% B (0.1% formic acid in acetonitrile, (Fisher Chemicals, Belgium)); 2.0–22.0 min, 5–35% B; 22.0–22.1 min, 35–80% B; 22.1–25.0 min, 80% B; 25.0–25.1 min, 80–85% B; 25.1–30.0 min, 5% B. Column temperature was kept at 40 °C. An information-dependent acquisition (IDA) method was set with a time-of-flight (TOF)-MS survey scan of 350–1,400 *m*/*z* for 50 ms. The 30 most intense precursors were selected for subsequent fragmentation, and the MS/MS were acquired in the range of 100–1,400 *m*/*z* for 25 ms, comprising a cycle time of 0.972 s. The data was acquired using Sciex OS software v. 3.1.6.44 (SCIEX, United States). External calibration was performed before each injection using the ESI Positive Calibration Solution (SCIEX, United States). Generated mass spectra (.wiff2 files) were converted to mzML format using the msconvert.exe tool from ProteoWizard (v.3.0.23111-67c7064). All searches were performed using MSFragger (v.4.1), Philosopher (v.5.1.1), and FragPipe (v.22.0). The protein database was created from the reviewed proteome of *A. acidoterrestris* DSM 3922^T^ (NCBI accession number CP080467) (downloaded on 07/2024, 4,004 entries) (Leonardo et al., 2022). Protein identification was considered with FDR <1%. Protein structure and biophysical characteristics of the Geo26 family candidate were analyzed using a combination of established bioinformatics tools. AlphaFold 3 was employed for high-accuracy three-dimensional structure prediction, while PyMOL (v.3.1.8) was used for structural visualization and analysis (Schrödinger, 2015; Jurrus et al., 2018; Ronneberger et al., 2021; Abramson et al., 2024). Physicochemical properties were assessed using ExPASy ProtParam (Gasteiger et al., 2005). Signal peptide prediction was performed with SignalP (v.5.0), and transmembrane regions were identified using TOPCONS (v.2.0), which integrates multiple prediction algorithms for improved reliability (Tsirigos et al., 2015; Almagro Armenteros et al., 2019). Sequence alignment and comparative analysis with Geo26 from *G. stearothermophilus* were conducted using MAFFT implemented in Geneious Prime (v2025.2.2), selected for its accuracy and efficiency in aligning homologous protein sequences (Katoh and Standley, 2013; Vaičikauskaitė et al., 2019).

### 2.3 Gene synthesis and *Escherichia coli* transformation

The Geo26 family protein identified in the *A. acidoterrestris* DSM 3922^T^ secretome was selected in this study as the candidate expected to exhibit antibacterial activity. To further investigate and characterize this protein, the codifying sequence of the Geo26 homolog, henceforth referred to as alicyclobacillin 24 or Ali24 for short, was synthesized with codon optimization for heterologous expression in *E. coli*. The signal peptide, corresponding to the first 27 amino acid residues as predicted using SignalP, was removed from the sequence (Almagro Armenteros et al., 2019). In addition, an Human Rhinovirus (HRV) 3C protease cleavage site followed by C-terminal 6xHis tag was introduced. This construct was cloned into the pET-21a(+) vector, which allows isopropyl β-D-1-thiogalactopyranoside (IPTG)-inducible gene expression. Gene synthesis and cloning were outsourced to NZYtech (Portugal), and the final nucleotide and amino acid sequences are presented in Supplementary Figure S1.

The recombinant plasmid pET-21a(+):Ali24, carrying an ampicillin resistance gene, was introduced by electroporation into *E. coli* Tuner™ (DE3) pLysS, which harbors a chloramphenicol resistance gene. *E. coli* transformants carrying the pET-21a(+):Ali24 construct were selected in presence of Cam_25_ and Amp_100_.

### 2.4 Heterologous expression of alicyclobacillin 24-His

A culture of *E. coli* Tuner™ (DE3) pLysS transformed with pET-21a(+):Ali24 was prepared by adding 1% (v/v) of an overnight pre-inoculum obtained from fresh colonies to 500 mL of fresh LB supplemented with Cam_25_ and Amp_100_. The liquid culture was incubated at 37 °C at 80 rpm in a New Brunswick Innova® 42R. When an optical density at 600 nm (OD_600nm_) of 0.6 was reached, the expression of the recombinant Ali24 (hereafter Ali24-His) was induced by the addition of 0.1 mM IPTG (Carl Roth, Germany) and the temperature was decreased to 18 °C. After 20 h, cells were harvested by centrifugation at 6,000g for 20 min at 4 °C in an Avanti J-26 XPI (Beckman Coulter, United States). The resulting pellets were saved at −70 °C until further use.

### 2.5 Protein purification and quantification

Each pellet was thawed and resuspended in 20 mL of purification buffer composed of 20 mM Bis-Tris propane (Melford, United Kingdom), 300 mM NaCl, and 20 mM imidazole (VWR, United States), pH 6.0, supplemented with 10 μg/mL DNase I (PanReac AppliChem, Germany) and 1 mM MgCl_2_. Cells were disrupted using a French Press (Thermo Scientific, United States) with 3 cycles at 1,000 psi. Cell debris was removed by centrifugation at 27,000g in a JA-25.50 rotor in an Avanti J-26 XPI for 90 min at 4 °C, and the purification of the Ali24-His was performed using a 5 mL HisTrap HP column (Cytiva, United States) equilibrated with the purification buffer. The elution was performed by applying a single step linear gradient with the same composition as the purification buffer, with an increased concentration of imidazole (300 mM and 500 mM imidazole for the first and second elution, respectively) over 20 column volumes Eliminate (CV) at a flow rate of 5 mL/min (Supplementary Figure S2). Eluted fractions were concentrated and subjected to buffer exchange using Amicon® Ultra centrifugal filters (Merck, United States) with a 10 kDa molecular weight cut-off, to remove imidazole and replace the purification buffer with a storage buffer composed of 50 mM citrate, 300 mM NaCl, pH 4.0. The retained protein concentration was determined using Nanodrop OneC (Thermo Fisher Scientific), correcting the 280 nm absorbance using the predicted extinction coefficient of the Ali24-His which corresponds to 1.642. To assess the purity of the eluted fractions and the concentrated protein, SDS-PAGE analysis was performed. Ali24-His was further analyzed by size exclusion chromatography (SEC) using a analytical Superdex 75 column 10/300 (GE Healthcare, United States), previously equilibrated with the storage buffer. Approximately 70 μg of recombinant protein was loaded onto the column and eluted at a flow rate of 0.3 mL/min. The oligomeric state was estimated by comparing its elution volume with a calibration curve generated with proteins of known MWs. The concentrated protein was stored in aliquots at 4 °C for immediate use, or at −70 °C for long-term storage.

### 2.6 Antibacterial activity assays

#### 2.6.1 Overlay agar assays to assess antibacterial potential

In this study, the antibacterial potential of different samples against the selected target bacteria was tested by overlay agar assays, using both spot plating and agar well diffusion formats. For this, fresh cultures of each target isolate were grown in BAT broth/LB and used to inoculate 5 mL of soft agar (BAT/LB supplemented with 0.5% (w/v) agar) with ∼10^6^ CFU/mL. The inoculated soft agar was poured onto the already solidified 20 mL of BAT agar/LA. After drying, samples were applied either as 10 μL drops (spot assay) or into 6 mm wells carved into the agar, prepared with a sterile UniCore Punch (QIAGEN), which were filled with 100 μL of sample. Plates were incubated at the optimal temperatures for each target isolate and examined after 24 h for the presence or absence of inhibition zones. Assays were performed in duplicate for every sample-target combination.

Double layer agar assays were first used to assess the antibacterial potential of *A. acidoterrestris* DSM 3922^T^ against the food isolate *A. acidoterrestris* MMB007, which has been previously characterized and explored as a susceptibility control isolate in studies involving ACB bacteria (Leonardo et al., 2026). In this case, a 24 h *A. acidoterrestris* DSM 3922^T^ culture and its respective supernatant, obtained after centrifuging the culture at 6,000g for 10 min, followed by filtration through a 0.45 μm PES syringe filter, were tested by spot plating.

To correlate Ali24-His expression with antibacterial activity, a lysate and its corresponding pellet of the transformed *E. coli* Tuner™ (DE3) pLysS pET-21(+):Ali24 were spot plated against a lawn of the susceptible target *A. acidoterrestris* MMB007. In parallel, as a vector control, a lysate and its corresponding pellet from *E. coli* Tuner pLysS pET-21a(+) culture were included (data not shown).

The same protocol was then applied to evaluate the antibacterial activity spectrum of Ali24-His, hence its specificity towards ACB species, by spot plating the recombinant protein onto lawns of a diverse panel of bacterial isolates. This included 19 ACB isolates representing five different species, strains from closely related genera such as *B. subtilis*, *B. cereus*, and *G. staerothermophilus*, as well as common hygiene indicator organisms in the food industry (*E. coli* and *S. aureus*) (Table 1). The storage buffer of Ali24-His was also tested as a negative control.

Finally, agar well diffusion assays were used to test the effect of temperature on Ali24-His antibacterial activity. For this, Ali24-His was incubated at 50 °C and 60 °C for 1 h and tested against a *A. acidoterrestris* MMB007 lawn using two-fold serial dilutions. A standard sample of the purified Ali24-His stored at 4 °C was also included.

#### 2.6.2 Microdilution method to determine minimum inhibitory concentration (MIC)

To determine the MIC for Ali24-His protein, antibacterial activity assays were performed following the broth microdilution method essentially according to the CLSI M07-A9 guidelines (CLSI, 2012), with the necessary medium and culture conditions adaptations. For this, *A. acidoterrestris* MMB007 and *A. acidoterrestris* MMB008 were selected as targets. The purified Ali24-His concentrated up to 5 mg/mL was distributed in a round-bottom microtiter 96-well plate, and 2-fold serial dilutions were prepared in BAT broth to obtain a range of concentrations for testing. The inoculum was prepared using the growth method to achieve a homogeneous suspension in saline solution. The adjusted inoculum was further diluted in BAT broth to guarantee that each well contained around 5 × 10^4^ CFU. The plates were incubated at 45 °C under aerobic conditions (ambient atmosphere, without active oxygenation) for 16 h to 20 h. For each assay, a positive control (BAT broth and diluted inoculum) and a medium sterility control (uninoculated BAT broth) were performed. A control of inoculated BAT with the Ali24-His storage buffer was also included. MIC values were selected as the lowest concentration of Ali24-His that visibly inhibited microbial growth after incubation and are presented as the median MIC of three independent assays.

#### 2.6.3 Membrane integrity assay

To determine whether Ali24-His induces membrane permeabilization in *A. acidoterrestris* MMB007, the LIVE/DEAD™ *Bac*Light™ Bacterial Viability Kit for microscopy (Thermo Fisher, United States) was used according to the manufacturer’s instructions. Briefly, an *A. acidoterrestris* MMB007 fresh culture was obtained by inoculating 1% (v/v) of an overnight culture, derived from pure colonies, into fresh BAT broth and incubating until mid-exponential phase (OD_600 nm_ between 0.6 and 0.8). Ali24-His was added to 1 mL of bacterial culture at the MIC value (1.25 mg/mL), and at each sampling point (0 min, 30 min, 1 h, 2 h, 4 h, and 6 h incubation), 100 μL were mixed with 0.1 μL of 3.34 mM SYTO 9 and 0.1 μL of 20 mM propidium iodide (PI). Samples were incubated in the dark for 2 min prior to imaging. Fluorescence visualization was performed using a Leica DM6000 B microscope (Leica Microsystems, Germany), and images were processed using ImageJ (v1.54p). To determine the percentage of permeabilized cells, approximately 200 to 300 cells per sample were counted over a minimum of 4 randomly selected microscope fields. Data is presented as the percentage of cells stained with PI, at each time point, as an average ± standard deviation (SD) of two independent assays. An untreated control, composed of *A. acidoterrestris* MMB007 not exposed to Ali24-His, was included.

### 2.7 Characterization of alicyclobacillin 24-His thermal properties

#### 2.7.1 Dynamic light scattering (DLS) analysis

To assess the temperature-dependent changes in Ali24-His homogeneity, the purified protein was first incubated at different temperatures, followed by DLS analysis using a SpectroLight 610 (Xtal Concepts GmbH, Germany). For this purpose, 20 μg of Ali24-His were incubated at 4 °C, 20 °C, 30 °C, 40 °C, 50 °C, 60 °C, 70 °C, 80 °C, and 90 °C for 1 h. After incubation, samples were centrifuged at 17,200g for 1 h at 4 °C. Two microliters of each sample were pipetted in duplicate onto the wells of a 96-well Vapor Batch Plate (Jena Bioscience GmbH, Germany). Before use, the plates were filled with paraffin oil (Merck, United States) to prevent the sample solutions from drying out. For measurement, a laser with a wavelength of 660 nm and a power of 100 mW was selected, and the scattering angle for placing the detector was fixed at 142 °. The refractive index (1.34) and viscosity (1.01) of water were used for calculations, as all tested samples were kept in the low-viscosity storage buffer. All samples were measured at a constant temperature of 20 °C, one scan per drop with 20 measurements of 20 s each.

#### 2.7.2 Nano differential scanning fluorimetry (NanoDSF)

To evaluate the pH-dependent effects on thermal stability of Ali24-His, the protein was first exposed to a range of pH conditions and subsequently analyzed by nanoDSF using a Prometheus NT.48 instrument (NanoTemper Technologies GmbH, Germany). For this, 2.5 μg of Ali24-His, maintained in the storage buffer, was mixed at a 1:2 (v/v) ratio with distinct 100 mM buffers from RUBIC additive screen buffers MD1-97 (Molecular Dimension, United Kingdom) and JBS solubility kit (Jena Bioscience, Germany): glycine pH 3.0, citrate pH 3.5, citrate pH 4.0, sodium acetate pH 4.5, citrate pH 5.0, citrate pH 5.5, potassium phosphate pH 6.0, MES pH 6.5, HEPES pH 7.0, sodium phosphate pH 7.5, BICINE pH 8.0, Tris-HCl pH 8.5, and CHES pH 9.0. Samples were incubated for 1 h at 4 °C prior to analysis. High-sensitivity capillaries (NanoTemper Technologies, Germany) were loaded with 10 μL of each sample and placed on the sample holder. A temperature gradient of 1 °C/min was applied from 20 °C to 95 °C, and the intrinsic protein fluorescence at 330 nm and 350 nm was recorded. Data was analyzed using the derived ratio 350 nm/330 nm value and melting temperatures (Tm) were determined from the midpoint of each transition, using the PR ThermControl software (v 2.16) and Stability Analysis software (v 1.1). All samples were tested in triplicate.

## 3 Results

### 3.1 Antibacterial activity of *Alicyclobacillus acidoterrestris* DSM 3922^T^

**Figure 1:**
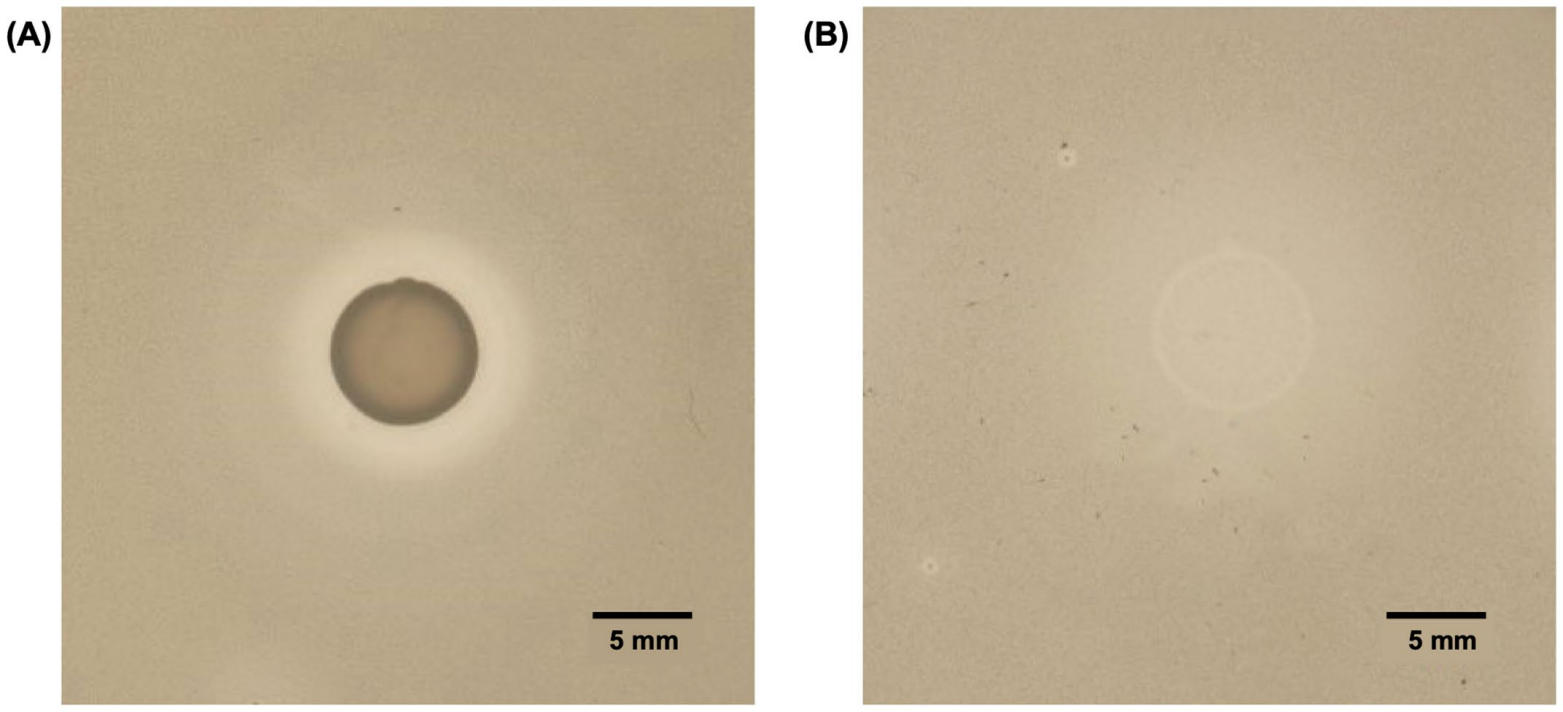
Antibacterial activity of *Alicyclobacillus acidoterrestris* DSM 3922^T^ against *A. acidoterrestris* MMB007, evaluated using the double-layer method. **(A)** Cultured cells produced a clear inhibition halo of approximately 2.75 mm, surrounded by a turbid halo of ~5 mm; **(B)** Culture supernatant generated a turbid spot of approximately 6 mm and a turbid halo of ~3.5 mm (Diameters and scale obtained by ImageJ).

To assess whether *A. acidoterrestris* DSM 3922ᵀ produces antibacterial compounds active against other ACB strains, its inhibitory activity was first assessed using the food isolate *A. acidoterrestris* MMB007 as target, selected based on its prior characterization and its use as a reference strain in susceptibility studies involving ACB bacteria (Leonardo et al., 2026). Both cultured cells and culture supernatant exhibited clear growth inhibition, as evidenced by the halos formed in agar overlay assays (Figure 1). Cultured cells produced a distinct clear halo of approximately 2.75 mm, surrounded by a turbid halo of ~5 mm (Figure 1A), whereas *A. acidoterrestris* DSM 3922^T^ supernatant formed a turbid spot of approximately 6 mm, followed by a turbid halo of ~3.5 mm (Figure 1B). The presence of a clearer halo for the cell-containing sample, compared with culture supernatant, likely reflects continuous production and secretion of the inhibitory compound during growth. These results indicate *A. acidoterrestris* DSM 3922^T^ secretes an extracellular bioactive compound capable of antagonizing closely related ACB isolates.

### 3.2 Proteomic analysis of *Alicyclobacillus acidoterrestris* DSM 3922^T^ secretome

Following confirmation that the culture supernatant of a 24 h culture of *A. acidoterrestris* DSM 3922^T^ retained antibacterial activity against *A. acidoterrestris* MMB007, the secretome of the type strain was concentrated and analyzed by LC-MS to identify compounds responsible for the observed inhibition. The proteomic analysis detected a total of 48 proteins in the concentrated supernatant of *A. acidoterrestris* DSM 3922^T^ (Supplementary Table S1). Of these, five proteins were excluded as they corresponded to trypsin, used in sample preparation, and human keratin, a common contaminant. The remaining 43 proteins were confidently identified and included transmembrane ATP-binding proteins, transporters, motility-related proteins (e.g., flagellin, flagellar hook-associated proteins), peptidoglycan-binding proteins, RNA-associated regulators (e.g., DEAD/DEAH box helicase), and metabolic enzymes (e.g., WrbA NADH:quinone oxidoreductase, S8 family serine peptidase). Eleven hypothetical proteins were also detected.

Among the proteins detected, only one candidate was predicted to possess antibacterial activity, a protein belonging to the Geo26 family, previously annotated in the *A. acidoterrestris* DSM 3922^T^ plasmid (locus tag K1I37_21410, sequence CP080468) (Leonardo et al., 2022). Six unique peptides aligned with the Geo26 family protein, corresponding to a sequence coverage of 46%, supporting its presence in the secretome and its secretion under the tested conditions.

Given its predicted function and secretion, the Geo26 family protein was selected as the most likely contributor to the antibacterial activity observed in *A. acidoterrestris* DSM 3922^T^. Based on its origin and predicted molecular weight of approximately 24 kDa, this protein was designated as alicyclobacillin 24 (Ali24).

### 3.3 Comparative analysis of alicyclobacillin 24 and geobacillin 26

To evaluate whether the Geo26 family protein identified in the *A. acidoterrestris* DSM 3922^T^ secretome was a plausible candidate for functional characterization, Ali24 was compared to the characterized Class III bacteriocin Geo26 from *G. stearothermophilus* through sequence- and structure-based analyses. Amino acid alignment performed with MAFFT revealed a low sequence identity of approximately 25% between Geo26 (245 amino acids) and its homolog identified in *A. acidoterrestris* DSM 3922^T^ (238 amino acids).

**Figure 2:**
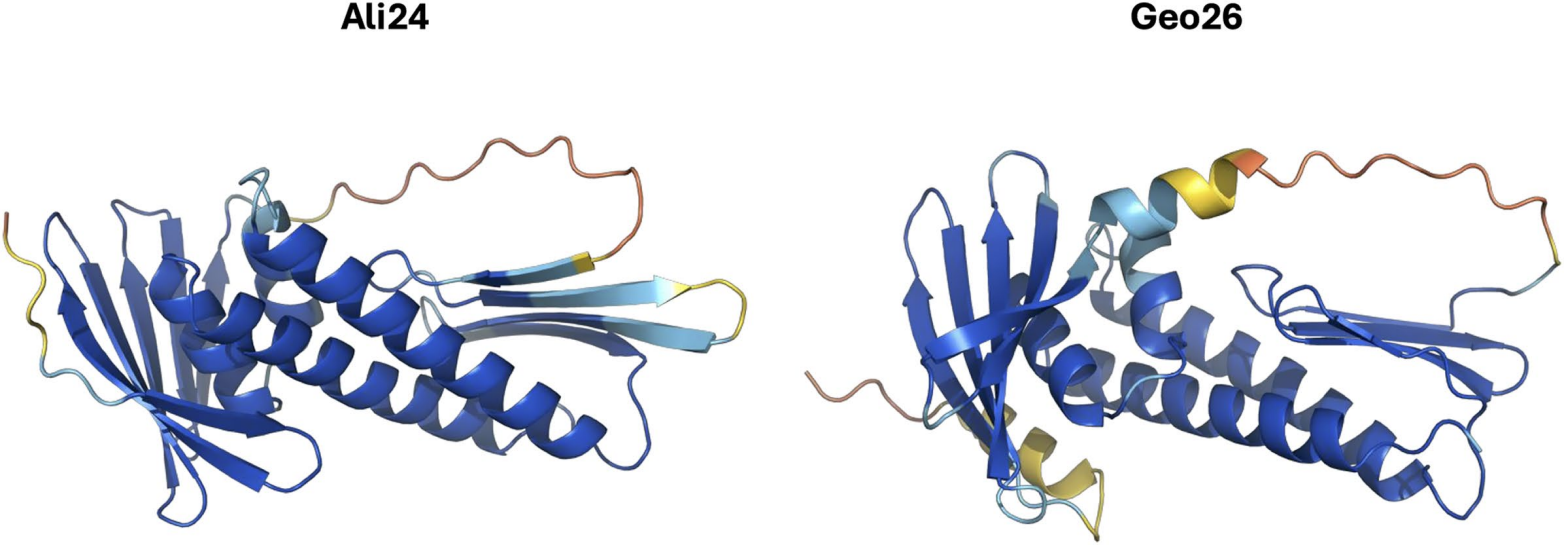
AlphaFold 3 structure prediction of alicyclobacillin 24 (Ali24) and geobacillin 26 (Geo26). Proteins are represented without the signal peptide regions predicted by SignalP.

Despite this limited sequence identity, both proteins share key structural and functional features typical of secreted antibacterial proteins. Signal peptide prediction using SignalP indicated that both Ali24 and Geo26 possess N-terminal secretory signal peptides engaging the Sec translocon and cleaved by the Signal Peptidase I (Sec/SPI) (Almagro Armenteros et al., 2019). These signal peptides comprise the first 27 residues in Ali24 and 25 residues in Geo26. Another shared feature between the two protein sequences is the presence of a putative C-terminal transmembrane helix. According to TOPCONS predictions, this domain is located between positions 205–225 in Geo26 and 198–218 in Ali24, with the predicted OUT → IN orientation indicating that the C-terminal region likely resides on the cytoplasmic side of the bacterial membrane (Tsirigos et al., 2015). In terms of overall structure, predictions generated using AlphaFold 3 produced confident models for both proteins, predicted template modeling (pTM) scores of 0.83 for Ali24 and 0.78 for Geo26 (Abramson et al., 2024), revealing comparable elongated architectures dominated by α-helical central cores supported by β-sheet elements (Figure 2).

Structural alignment between Ali24 and Geo26 revealed substantial conservation of the overall fold, particularly across core domains, despite variability in flexible regions and terminal segments (Supplementary Figure S3).

### 3.4 Heterologous expression and purification of alicyclobacillin 24-His

Ali24-His was heterologously expressed in *E. coli* and purified in quantities sufficient for subsequent antibacterial activity assays and biophysical characterization. Since the native protein from *A. acidoterrestris* DSM 3922^T^ is secreted and active under acidic conditions (pH 4.0), the purification protocol was optimized to maintain a low pH, which is expected to support bacteriocin stability and activity. His-tag affinity purification was performed at pH 6.0, lower than standard conditions, and the purity of the eluted fractions was confirmed by SDS-PAGE, showing a single band at approximately 25 kDa, consistent with the predicted MW and with no detectable contaminants (Supplementary Figure S4). The purified protein was subsequently concentrated and buffer-exchanged into the storage buffer at pH 4.0 for downstream activity and stability assays. Using this strategy, Ali24-His was obtained at a yield of approximately 45 mg per liter of culture.

**Figure 3:**
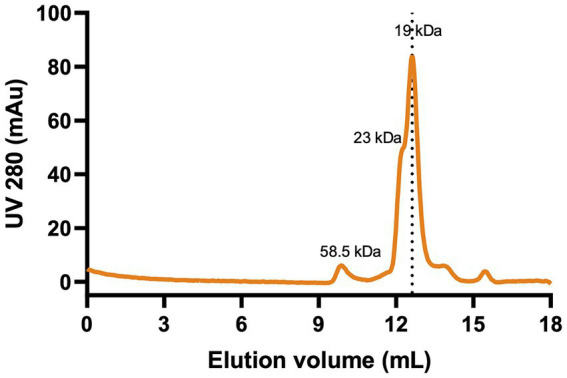
Size-exclusion chromatography profile of recombinant alicyclobacillin 24-His. Chromatography was carried out on a Superdex 75 10/300 GL. Molecular weight of oligomeric forms were estimated from calibration curve prepared with a protein gel filtration molecular weight marker (Low Molecular Weight Kits with range of 6,500 to 75,000 Da). The protein eluted mainly as a monomer (~90%), with a smaller peak corresponding to its dimeric form.

The oligomeric state and homogeneity of purified Ali24-His were examined by SEC (Figure 3). Approximately 90% of the protein eluted at a volume corresponding to the monomeric state, while a minor fraction eluted at a volume consistent with a dimer state. The main peak exhibited slight peak broadening, which may reflect conformational flexibility of Ali24-His in solution or deviations from globular geometry of the protein, consistent with its elongated structure (Figure 2) (Rambo, 2017). Overall, the SEC profile indicates that Ali24-His is homogeneous, suggesting it is well suited for subsequent functional and stability analyses.

### 3.5 Antibacterial activity of recombinant alicyclobacillin 24-His

#### 3.5.1 Antibacterial activity spectrum

To assess the antibacterial activity spectrum of Ali24-His, overlay agar assays were conducted against a diverse panel of 24 bacterial isolates, including 19 strains belonging to the ACB genus and five representatives from other Gram-positive and Gram-negative genera (Table 1). The Ali24-producing strain *A. acidoterrestris* DSM 3922^T^ was included as a negative control, as no antibacterial activity was expected nor detected (Table 2).

**Table 2:** Antibacterial activity of recombinant alicyclobacillin 24-His (Ali24-His) against other bacteria.

Bacterial strain	Antibacterial activity
*A. acidoterrestris* DSM 3922^T*^	−
*A. acidiphilus* DSM 14558^T^	+
*A. acidocaldarius* DSM 446^T^	+
*A. acidocaldarius* MMB020	+
*A. acidoterrestris* MMB001	+
*A. acidoterrestris* MMB002	+
*A. acidoterrestris* MMB003	−
*A. acidoterrestris* MMB004	+
*A. acidoterrestris* MMB005	+
*A. acidoterrestris* MMB006	+
*A. acidoterrestris* MMB007	+
*A. acidoterrestris* MMB008	+
*A. acidoterrestris* MMB009	+
*A. acidoterrestris* MMB010	+
*A. acidoterrestris* MMB011	+
*A. acidoterrestris* MMB012	+
*A. acidoterrestris* MMB013	+
*A. acidoterrestris* MMB021	−
*A. cycloheptanicus* DSM 4006^T^	+
*A. fastidiosus* DSM 17978^T^	+
*B. subtilis* CECT 356	−
*B. cereus* BGSC 6A1	−
*E. coli* ATCC 25922	−
*G. stearothermophilus* DSM 22^T^	−
*S. aureus* ATCC 6538	−

+: inhibition zones were observed; −: no inhibition was observed.

^*^Ali24-producing strain, included as a negative control.

Ali24-His exhibited a narrow-spectrum activity, specific towards ACB isolates, as no inhibitory effect was observed against other Gram-positive and Gram-negative strains tested (Table 2). Among the 19 ACB isolates tested, Ali24-His successfully inhibited the growth of 17 isolates, including all five species examined. Inhibition patterns varied across isolates tested, ranging from clear to more turbid halos, and reflecting Ali24-His concentration-dependent effect or a more constant inhibitory zone, consistent with a bacteriostatic activity (Supplementary Figure S5).

#### 3.5.2 Minimum inhibitory concentration

Further characterization of Ali24-His included determination of its MIC against *A. acidoterrestris* MMB007 and *A. acidoterrestris* MMB008, selected because they exhibited different inhibition patterns in the overlay agar assays described above, with *A. acidoterrestris* MMB007 showing more turbid inhibition halos than *A. acidoterrestris* MMB008, which exhibited a clear inhibition halo, independent of Ali24-His concentration (Supplementary Figure S6). Ali24-His displayed a MIC of 1.25 mg/mL against *A. acidoterrestris* MMB007 and 19.5 μg/mL against *A. acidoterrestris* MMB008, supporting an association between clearer inhibition halos and lower MIC values, while highlighting the pronounced strain-dependent variability in susceptibility to Ali24-His.

#### 3.5.3 Membrane integrity

**Figure 4:**
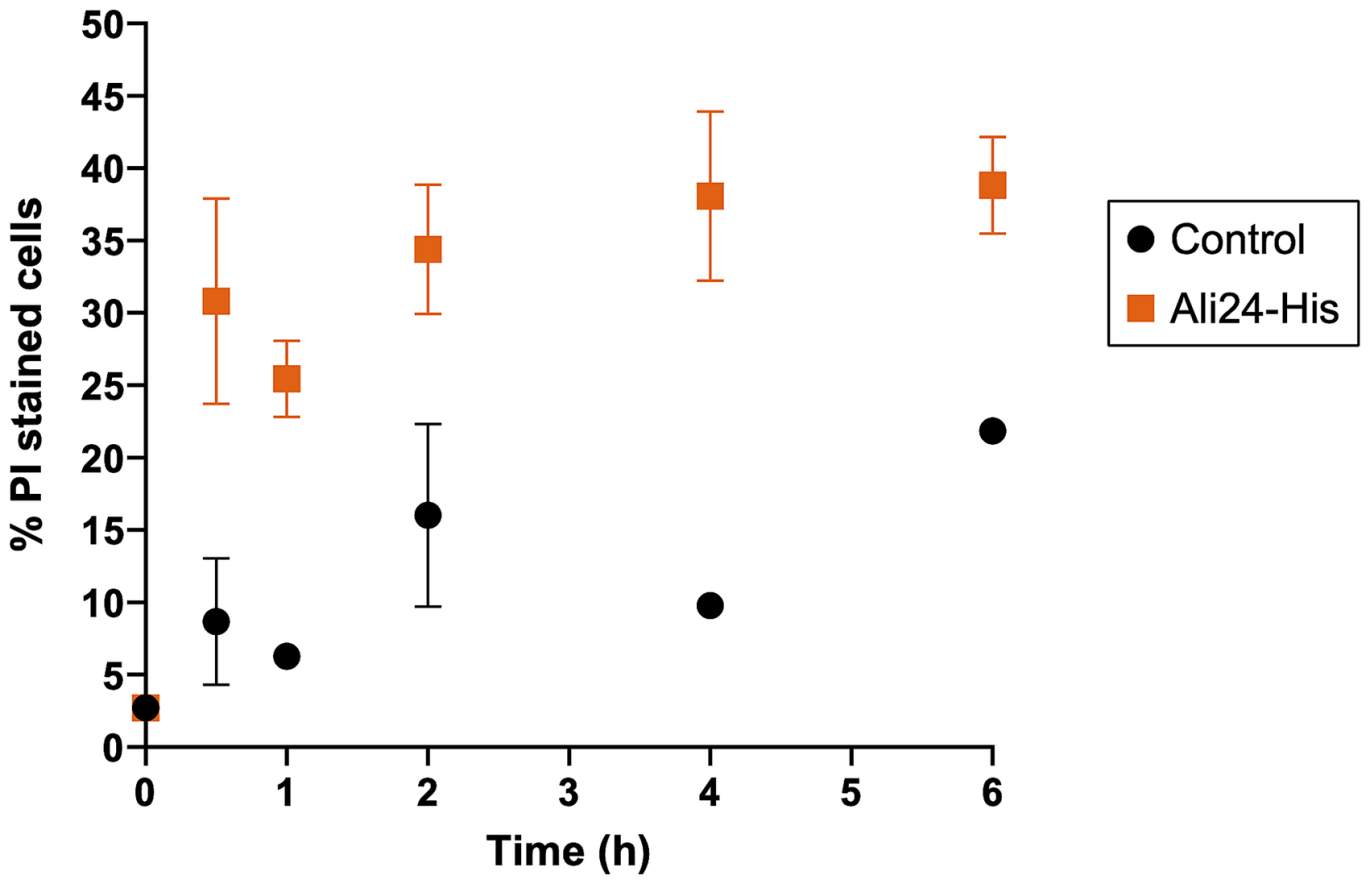
Membrane damage of *Alicyclobacillus acidoterrestris* MMB007 exposed to the recombinant alicyclobacillin 24-His (Ali24-His). *A. acidoterrestris* MMB007 cultures were exposed to Ali24-His at its minimum inhibitory concentration (1.25 mg/mL, orange squares) or left untreated (black circles, control), and at the indicated times cells were stained with SYTO9 and propidium iodide (PI) and examined by fluorescence microscopy to quantify membrane-compromised cells. Data is shown as mean ± standard deviation of two independent experiments, in which approximately 200–300 cells per sample were counted across at least four randomly selected microscopic fields.

In addition, the effect of Ali24-His on bacterial membrane integrity was assessed using a fluorescence-based membrane integrity assay based on SYTO9 and PI staining. Exposure of *A. acidoterrestris* MMB007 to the MIC of Ali24-His led to a time-dependent increase in the proportion of PI-stained cells compared with the untreated control culture, indicating that exposure to this protein is associated with a progressive loss of membrane integrity in the target bacterium (Figure 4). The effect becomes evident early after exposure, with an increase of approximately 20% in permeabilized cells after 30 min exposure (Figure 4). This effect persists at later time points, where the percentage of PI-stained cells in the treated samples clearly exceeds that of the control up to 6 h, suggesting that membrane damage accumulates during incubation with Ali24-His.

**Figure 5:**
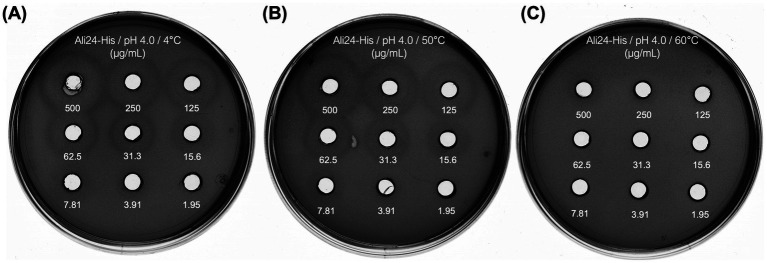
Antibacterial activity of the recombinant alicyclobacillin 24-His (Ali24-His) against *Alicyclobacillus acidoterrestris* MMB007 following exposure to acidic and thermal conditions. Agar well diffusion assays testing Ali24-His exposed to **(A)** pH 4.0, 4 °C, **(B)** pH 4.0, 50 °C, and **(C)** pH 4.0, 60 °C.

To obtain preliminary information relevant to the potential use of Ali24 in ACB-containing food matrices, its antibacterial activity was assessed under industry-relevant conditions. Given its previously demonstrated effectiveness at acidic pH, Ali24-His was tested after exposure to higher temperatures. Specifically, Ali24-His was exposed to 50 °C and 60 °C for 1 h at pH 4.0, and its activity was compared with samples maintained under standard storage conditions (pH 4.0 and 4 °C) serving as a control, enabling a direct assessment of how thermal stress influence antimicrobial function. Analysis of inhibition halos from serial dilutions of Ali24-His showed that this protein retained full antibacterial activity after exposure to 50 °C, with results comparable to the control (Figures 5A,B). In contrast, exposure to 60 °C (Figure 5C) significantly impairs its antimicrobial activity. This is evidenced by the complete absence of inhibition against *A. acidoterrestris* MMB007, even at the highest protein concentration tested.

### 3.6 Biophysical stability of alicyclobacillin 24-His under thermal and pH stress

To further evaluate the suitability of Ali24-His as an alternative food preservative targeting ACB bacteria, its stability under industrially relevant thermal and pH stress conditions was assessed.

**Figure 6:**
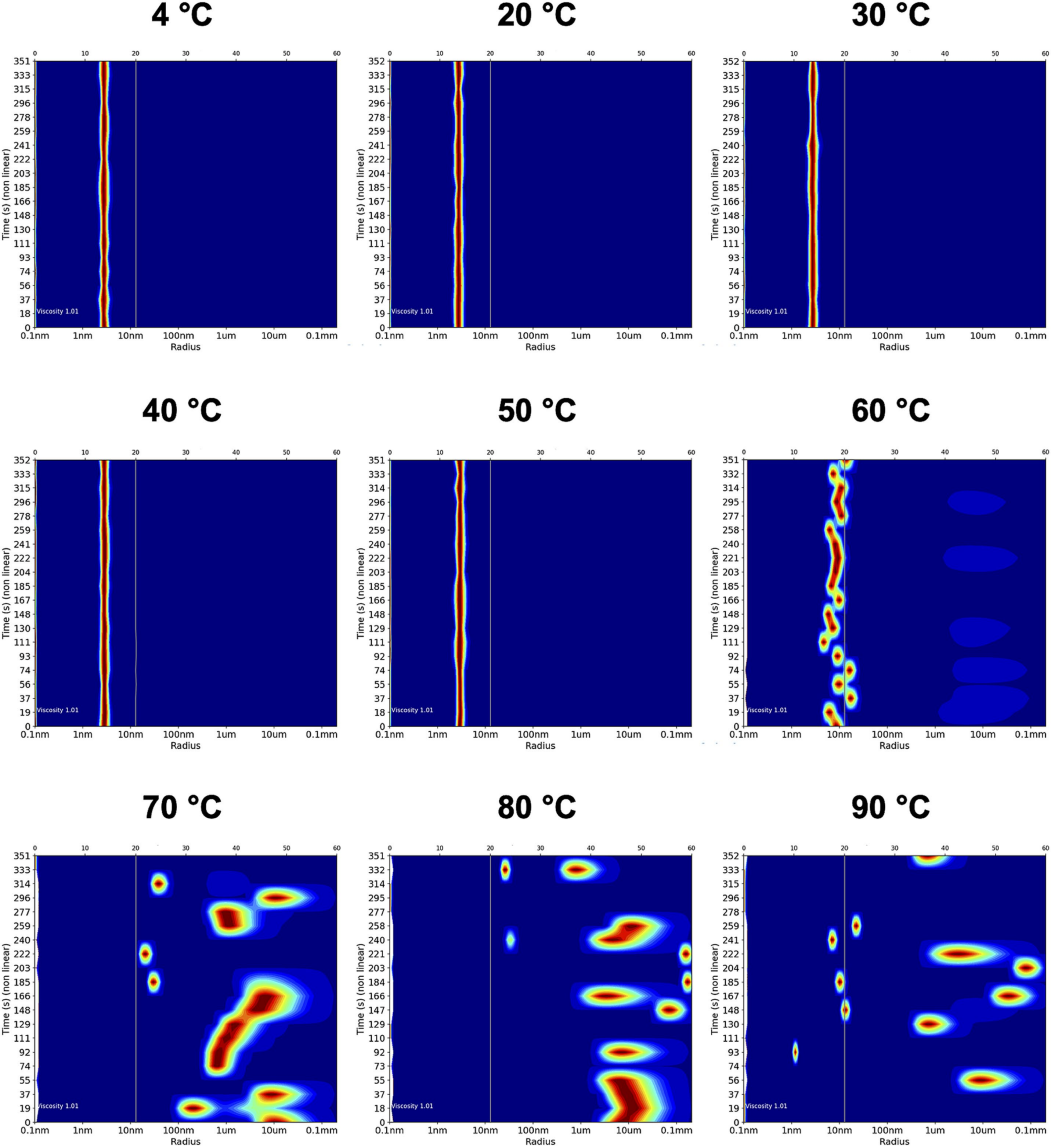
Dynamic light scattering heat map analysis of the recombinant alicyclobacillin 24-His after 1 h incubation at different temperatures. Two independent measurements were performed, and a representative of each temperature is presented.

In this study, the temperature-dependent effects on Ali24-His homogeneity were assessed by DLS following exposure to increasing temperatures under conditions relevant to fruit processing, including pasteurization steps, as heat-induced heterogeneity and aggregation usually correlates with loss of functionality. The DLS profiles of Ali24-His revealed homogeneous and monodisperse distributions up to 50 °C, indicating that the recombinant protein remains well-folded and resistant to aggregation under these conditions (Figure 6, Table 3). Samples incubated at temperatures between 4 °C and 50 °C exhibited consistent polydispersity index (PDI) values of approximately 12%, indicative of monodisperse samples (Bhattacharjee, 2016), and predicted MW of ~37 kDa. The apparent MW was approximately 12 kDa higher than the theoretical MW of Ali24-His, consistent with deviations from the globular behavior assumed in DLS analysis for elongated protein architectures (Stetefeld et al., 2016). At 60 °C, a slight increase of the PDI was observed, accompanied by a substantial overestimation of the MW of Ali24-His, consistent with the onset aggregation (Figure 6, Table 3). Temperatures above 70 °C completely destabilized the protein, preventing reliable determination of PDI or MW (Figure 6, Table 3). Overall, these results indicate that Ali24-His remains stable and resistant to aggregation up to 50 °C, which is consistent with the previously demonstrated antibacterial activity up to the same temperature.

**Table 3:** Dynamic light scattering measurements of the recombinant alicyclobacillin 24-His after 1 h induction at different temperatures.

Condition	PDI (%)	MW (kDa)
4 °C	11.75 ± 0.05	36.24 ± 0.66
20 °C	11.4 ± 0.10	36.18 ± 0.25
30 °C	11.75 ± 0.25	36.57 ± 0.56
40 °C	11.9 ± 0.20	37.58 ± 0.12
50 °C	12.25 ± 0.15	41.61 ± 0.11
60 °C	16.45 ± 0.75	756.1 ± 278
70 °C	NR	NR
80 °C	NR	NR
90 °C	NR	NR

Polydispersity index (PDI) and molecular weight (MW) values represent the mean ± standard deviation from 2 independent measurements.

NR - not reliably determined due to aggregation.

**Figure 7:**
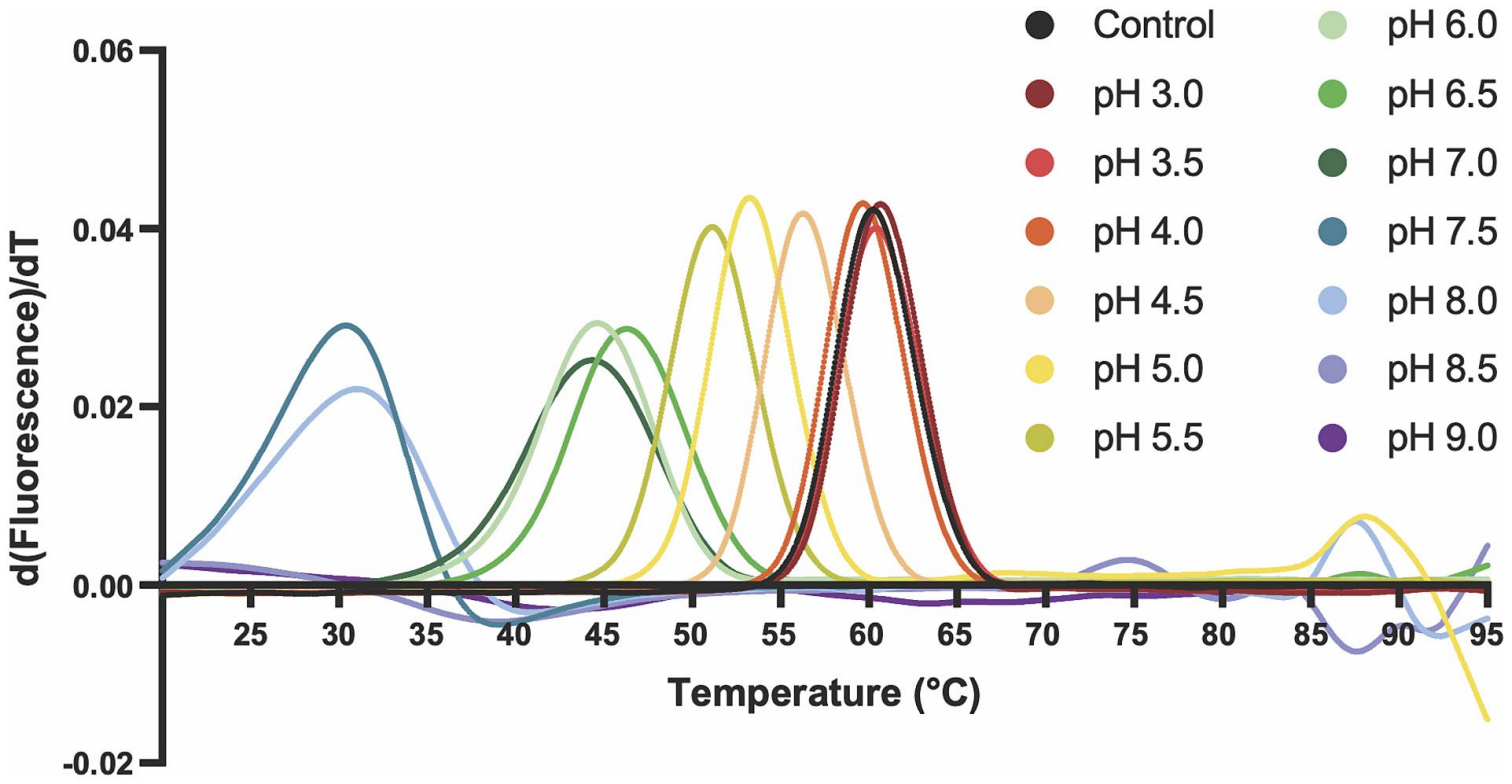
NanoDSF thermal unfolding profiles of the recombinant alicyclobacillin 24-His in buffers ranging from pH 3 to pH 9. The derived fluorescence ratio (350 nm/330 nm) was monitored as a function of temperature to assess protein stability across 14 different pH conditions. Each curve represents a distinct buffer pH, color-coded from red (acidic) to purple (basic) to reflect the pH gradient. Data are representative of three independent experiments.

The influence of pH on Ali24-His thermal stability was evaluated by nanoDSF, monitoring temperature-induced unfolding across a wide range of pH values, from pH 3 to pH 9, covering the acidic conditions typically encountered in fruit processing as well as neutral and mildly basic environments relevant to other applications. Ali24-His exhibited its highest thermal stability under acidic conditions, with consistent Tm values above 50 °C between pH 3.0 and pH 5.5 (Figure 7, Table 4). As pH increased towards neutrality (pH 6.0 to pH 7.0), a gradual decrease in Tm was observed. Thermal stability dropped sharply above pH 7.5, where unfolding occurred at approximately 30 °C (pH 7.5 to pH 8.0) or could not be reliably determined (pH 8.5 and above) (Figure 7, Table 4). At the control condition (pH 4.0), the determined Tm was 60.4 °C, in agreement with DLS measurements, showing that Ali24-His destabilization and aggregation occurred only above 50 °C.

**Table 4:** Melting temperatures (Tm) of the recombinant alicyclobacillin 24-His determined by nanoDSF across a range of buffer pH values.

Sample buffer	Tm (°C)	ΔTm (°C)
Control (Storage buffer - citrate, NaCl pH 4.0)	60.4 ± 0.15	—
Glycine pH 3.0	60.8 ± 0.04	0.4
Citrate pH 3.5	60.5 ± 0.01	0.1
Citrate pH 4.0	59.7 ± 0.03	−0.7
Sodium acetate pH 4.5	56.4 ± 0.02	−4
Citrate pH 5.0	53.3 ± 0.07	−7.1
Citrate pH 5.5	51.2 ± 0.09	−9.2
Potassium phosphate pH 6.0	44.6 ± 0.01	−15.8
MES pH 6.5	46.3 ± 0.05	−14.1
HEPES pH 7.0	44.3 ± 0.02	−16.1
Sodium phosphate pH 7.5	30.2 ± 0.01	−30.2
BICINE pH 8.0	30.8 ± 0.08	−29.6
Tris-HCl pH 8.5	ND	ND
CHES pH 9.0	ND	ND

Tm values were extracted from the inflection point of the unfolding curves and represent the mean ± standard deviation from three independent measurements. The shift of the melting point towards the control setting is represented as ΔTm in degree Celsius.

## 4 Discussion

The antibacterial activity exhibited by *A. acidoterrestris* DSM 3922ᵀ indicates that this spoilage strain secretes extracellular inhibitory compounds. This finding prompted a targeted analysis of the *A. acidoterrestris* DSM 3922^T^ secretome to identify the secreted factors associated with the observed inhibitory phenotype and to assess whether the predicted Geo26-like protein contributed to this activity (Leonardo et al., 2022).

Proteomic analysis confirmed the secretion of a Geo26-like protein (Ali24), supporting that *A. acidoterrestris* DSM 3922^T^ expresses a bacteriocin under standard culture conditions. Its substantial sequence coverage, combined with the absence of other predicted antimicrobial proteins in the secretome, strongly suggests that Ali24 is the primary contributor to the observed antagonistic activity.

Ali24 shares several hallmark features with Geo26, a Class III bacteriocin, including a Sec-dependent secretion signal peptide, comparable molecular sizes, a predicted C-terminal transmembrane helix, and a conserved elongated α-helical core, despite exhibiting low primary sequence identity. Similar cases of structural conservation regardless of limited primary sequence similarity have been described in other bacteriocin families, such as circular bacteriocins, where fold architecture rather than amino acid identity determines bioactivity (Acedo et al., 2018). These structural and functional similarities support the classification of Ali24 as a Geo26-like Class III bacteriocin and justified its heterologous expression and functional characterization as the first bacteriocin described from an ACB species. All functional analyses were conducted using the C-terminal His-tagged recombinant form (Ali24-His). Although minor effects of the tag on absolute activity or stability cannot be completely excluded, the robust antibacterial activity, strain specificity, and overall biophysical behavior observed are expected to reflect the properties of the native protein. Furthermore, C-terminal His-tags are widely used for bacteriocin and antimicrobial protein characterization and typically do not alter their qualitative activity profiles.

Mechanistic analysis showed that Ali24-His exposure resulted in a progressive increase in PI-positive cells, supporting a membrane-associated mode of action. This behavior is in agreement with the predicted C-terminal transmembrane domain in Ali24, similar to that of Geo26, and mirrors the membrane alterations described for Geo26, which causes surface irregularities without apparent pore formation (Vaičikauskaitė et al., 2019).

Ali24-His exhibited a narrow antibacterial spectrum restricted to ACB species, which is consistent with the genus-level specificity commonly observed among Class III bacteriocins (Vaičikauskaitė et al., 2019; Antoshina et al., 2022; Yi et al., 2022). Inhibition patterns varied between isolates, with some profiles compatible with bacteriostatic activity. These results demonstrate that Ali24-His targets multiple species frequently associated with spoilage of acidic fruit-based products, reinforcing its potential relevance within the context of spoilage microbiology (Cai et al., 2025b).

Ali24-His exhibited a notable stability under acidic pH and moderate temperatures, reflecting the microenvironment inhabited by ACB and the conditions typical of early fruit-processing steps. Structural and functional stability under these conditions is a key requirement for Ali24-His potential use as a biopreservative, and it often correlates with resistance to chemical degradation and greater tolerance to other destabilizing factors (Stetefeld et al., 2016). Overall, the activity and stability profiles of Ali24-His support its potential as a targeted antimicrobial candidate for controlling ACB-related spoilage in acidic food processing environments. Ali24 can be further explored for integration into earlier fruit processing steps, prior to pasteurization, or storage that occurs below structural and activity destabilization threshold temperatures (above 50 °C).

Ali24-His specificity against different spoilage-related ACB and its strain-dependent inhibitory efficacy suggests that this antibacterial protein may contribute to competitive interactions in acidic food-associated environments. In such ecosystems, where thermoacidophilic spore-formers coexist under selective pressures imposed by low pH and thermal treatments, bacteriocin production may represent an adaptive strategy to secure ecological fitness. By limiting the growth of closely related competitors, Ali24 could contribute to niche stabilization and community structuring within ACB populations. The ability to remain structurally stable and functionally active under acidic and thermally dynamic conditions may further extend the window of antimicrobial activity, allowing bacteriocin-producing strains to sustain competitive pressure within fluctuating food-associated environments. Such resilience may reflect an adaptive trait enabling thermoacidophilic bacteria to maintain ecological fitness in acidic food ecosystems characterized by repeated thermal and pH stresses, while also supporting a possible relevance for future technological applications.

Despite these promising findings, several limitations remain. As PI staining alone does not distinguish between the lytic and non-lytic activity or reveal the precise molecular mechanism, additional assays such as membrane potential assays, ion-leakage measurements, or high-resolution imaging will be required to elucidate the molecular mechanism underlying Ali24 activity. In addition, further studies are required to evaluate its efficacy within relevant food matrices, and assess safety parameters in accordance with established regulatory frameworks (EFSA Panel on Food Additives and Flavourings (FAF) et al., 2026).

Bacteriocins are widely regarded as promising natural alternatives to synthetic preservatives due to their specificity and biodegradability, and numerous candidates have been described with potential applications in food systems (Belguesmia et al., 2020; Pei et al., 2020; Lozo et al., 2021; Soltani et al., 2021; Xiang et al., 2022). However, despite the large number described, only nisin and pediocin PA-1 have reached commercial approval by the European Food Safety Authority (EFSA) and the Food and Drug Administration (FDA), highlighting the importance of rigorous and standardized characterization. Within this broader context, Ali24 expands the repertoire of bacteriocins derived from thermoacidophilic bacteria and represents a promising candidate for further investigation in the framework of sustainable spoilage control strategies.

## 5 Conclusion

The capacity of spoilage-associated ACB to tolerate acidic environments and thermal processing highlights the need for alternative preservation strategies in acidified food systems. In this context, the present study provides new evidence that *A. acidoterrestris* DSM 3922^T^ encodes and secretes a Geo26-like protein, here designated Ali24, the first Class III bacteriocin. Functional characterization showed that Ali24-His exhibits a narrow antibacterial spectrum targeting multiple spoilage-associated ACB species and retains activity under acidic pH and moderate temperature conditions relevant to food processing environments. These findings provide new insight into antimicrobial strategies that may shape competitive interactions within acidic environments associated with spoilage ACB and highlight thermoacidophilic bacteria as an underexplored source of bacteriocins.

Altogether, Ali24 represents a promising addition to the limited repertoire of bacteriocins derived from thermoacidophilic bacteria. Its activity and stability under relevant processing conditions support its potential for targeted control of ACB-related spoilage. Future work should focus on elucidating its mechanism of action, validating its performance in real food systems, and assessing its safety and regulatory suitability, paving the way for its potential application in sustainable food preservation strategies.

## Data Availability

The datasets presented in this study can be found in online repositories. The names of the repository/repositories and accession number(s) can be found in the article/Supplementary material.
