# Supplementary material for: Alicyclobacillin 24: a class III bacteriocin from Alicyclobacillus acidoterrestris targeting species associated with spoilage of acidic fruit-based products
Source: Front Microbiol. 2026 May 1;17:1823210. doi: 10.3389/fmicb.2026.1823210 (PMC13176240; doi:10.3389/fmicb.2026.1823210)
Supplement: Supplementary file 1 [file presentation_1.zip › Supplementary Material Figure S1.docx]

Supplementary Material


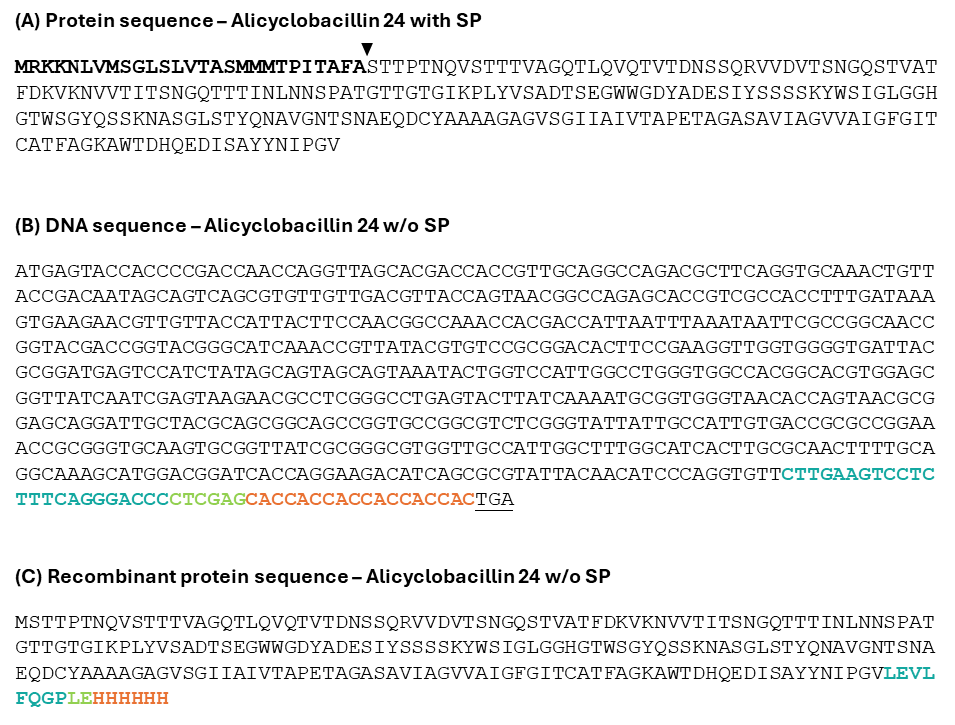


**Supplementary Figure S1.** Alicyclobacillin 24 nucleotide and amino acid sequences. (A) Protein sequence retrieved from *A. acidoterrestris* DSM 3922^T^ proteome. The signal peptide is presented in bold, and the arrow marks the cleavage site predicted by SignalP 5.0; (B) Nucleotide sequence of alicyclobacillin 24 without the signal peptide, with a HRV (3C) site (blue), a XhoI recognition site (green), a C-terminal 6xHis tag (orange), and the STOP codon (underlined); (C) final recombinant protein sequence, with HRV (3C) site (blue), XhoI recognition site (green) and C-terminal 6xHis tag (orange).
