# Supplementary material for: Alicyclobacillin 24: a class III bacteriocin from Alicyclobacillus acidoterrestris targeting species associated with spoilage of acidic fruit-based products
Source: Front Microbiol. 2026 May 1;17:1823210. doi: 10.3389/fmicb.2026.1823210 (PMC13176240; doi:10.3389/fmicb.2026.1823210)
Supplement: Supplementary file 1 [file presentation_1.zip › Supplementary Material Figure S3.docx]

Supplementary Material


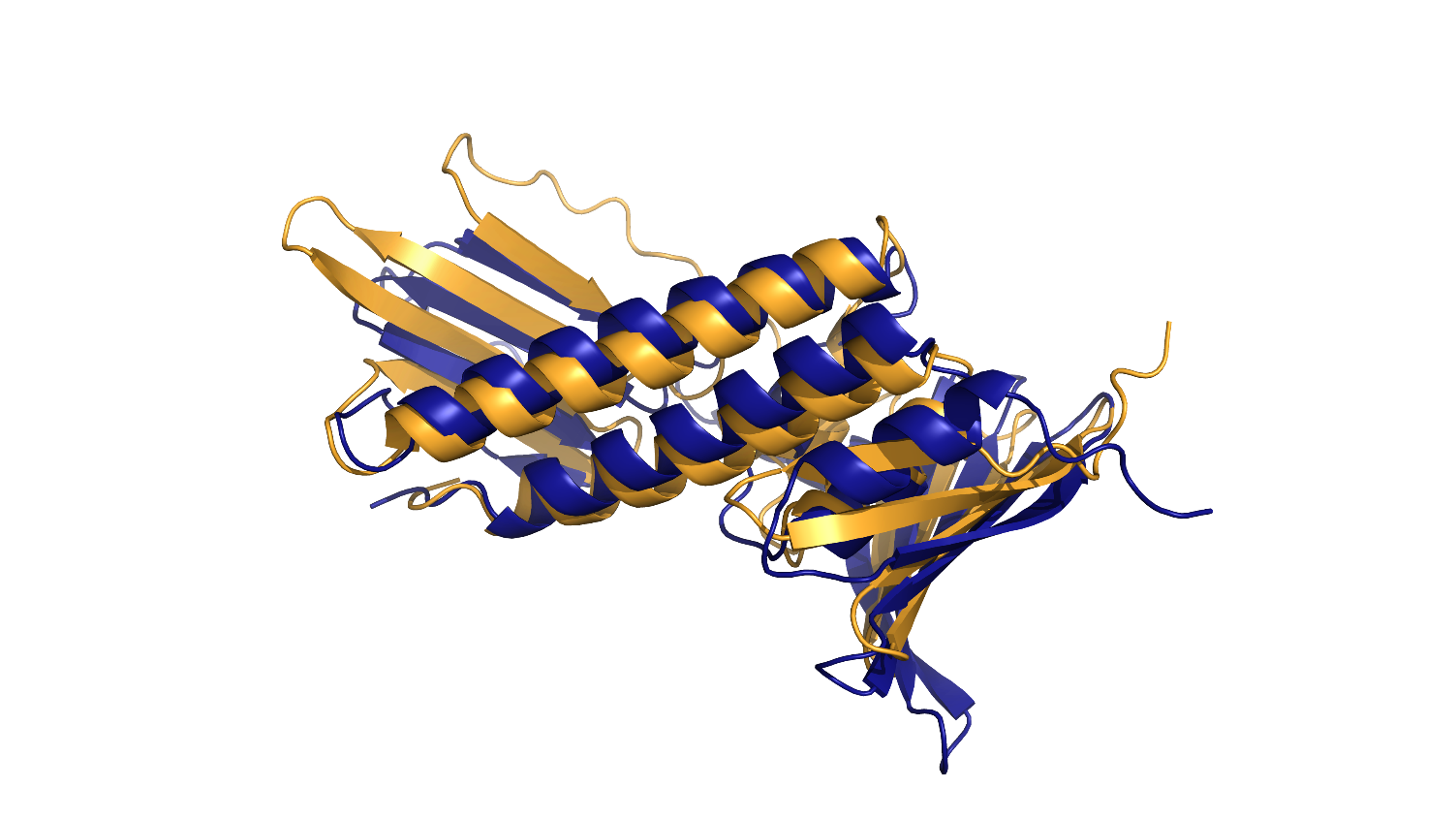


**Supplementary Figure S3.** Alignment of Ali24 and Geo26 structural predictions. Results obtained in Pymol, using structural predictions of alphaFold 3. Structural alignment reveals conservation of core regions composed of alpha helices and beta sheets, with local deviations in flexible loops, terminal regions, or domain orientation, as reflected in the high root-mean-square deviation (RMSD) calculated of 4.5 Å using Pymol.
