# Supplementary material for: Alicyclobacillin 24: a class III bacteriocin from Alicyclobacillus acidoterrestris targeting species associated with spoilage of acidic fruit-based products
Source: Front Microbiol. 2026 May 1;17:1823210. doi: 10.3389/fmicb.2026.1823210 (PMC13176240; doi:10.3389/fmicb.2026.1823210)
Supplement: Supplementary file 1 [file presentation_1.zip › Supplementary Material Figure S4.docx]

Supplementary Material


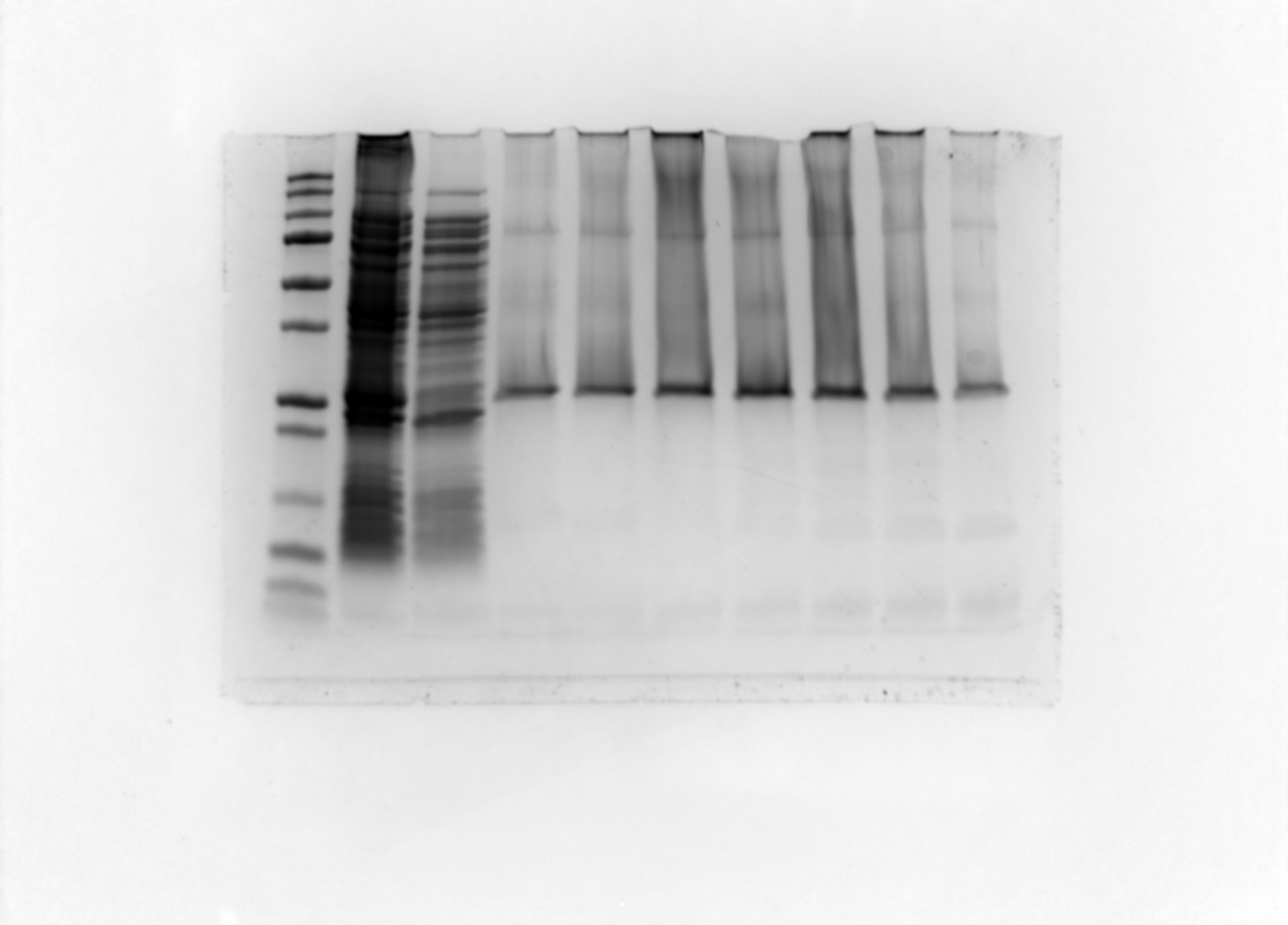

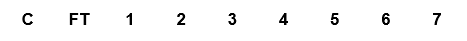

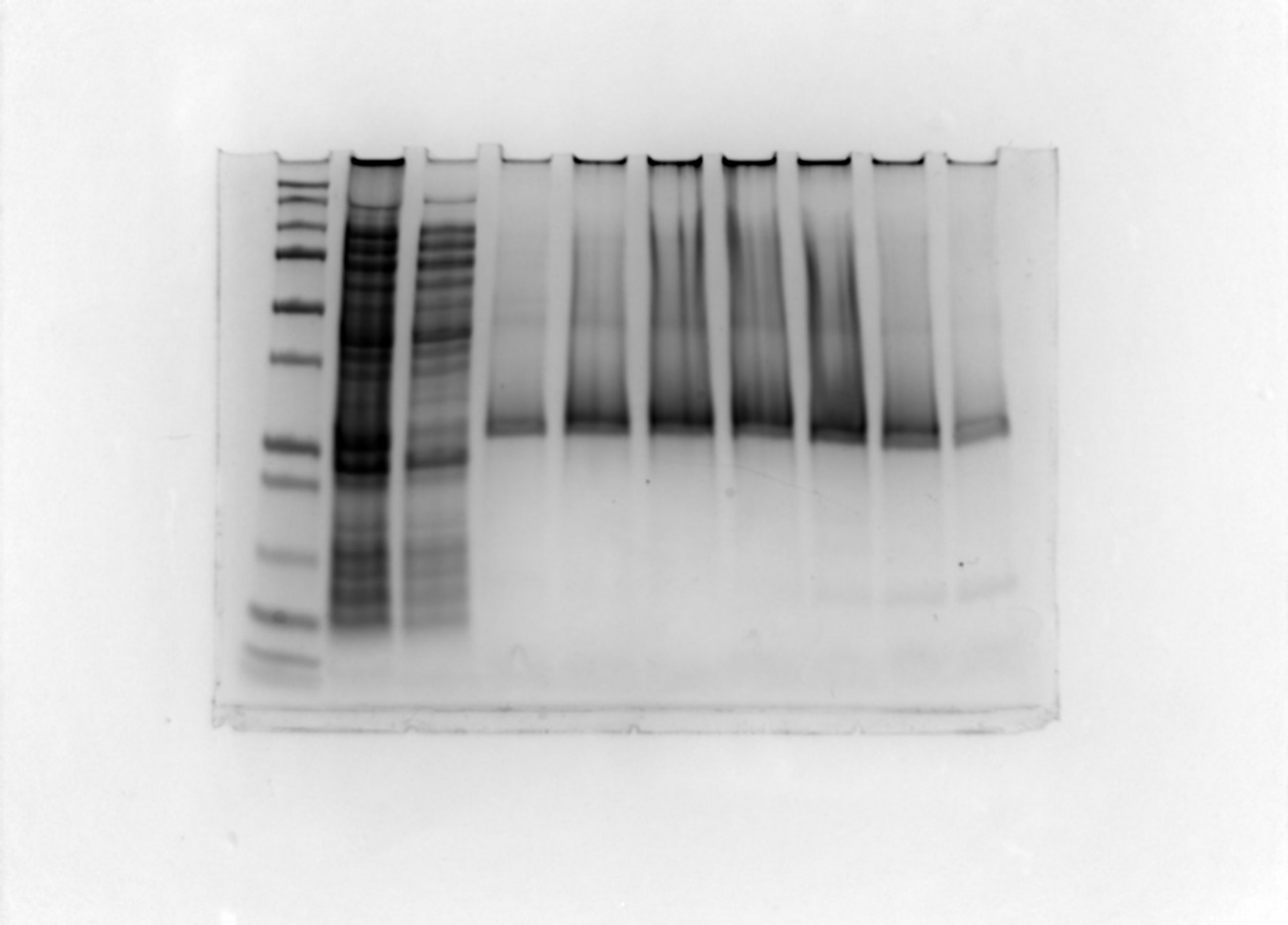

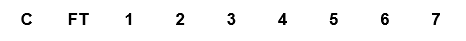


250 kDa

150 kDa

100 kDa

75 kDa

50 kDa

37 kDa

25 kDa

20 kDa

15 kDa

10 kDa

250 kDa

150 kDa

100 kDa

75 kDa

50 kDa

37 kDa

25 kDa

20 kDa

15 kDa

10 kDa

**(A)**

**(B)**

**Supplementary Figure S4**. SDS-PAGE analysis of Ali24-His purified by HisTrap affinity chromatography column. Fractions collected during the elution are shown for (A) the first injection and (B) the second injection. In both SDS-PAGE, the first lane contains the Precision Plus Protein Ladder (Bio-Rad, USA), followed by samples corresponding to C – crude extract, FT – flowthrough, and fractions 1–7 collected during elution.
