# Supplementary material for: Alicyclobacillin 24: a class III bacteriocin from Alicyclobacillus acidoterrestris targeting species associated with spoilage of acidic fruit-based products
Source: Front Microbiol. 2026 May 1;17:1823210. doi: 10.3389/fmicb.2026.1823210 (PMC13176240; doi:10.3389/fmicb.2026.1823210)
Supplement: Supplementary file 1 [file presentation_1.zip › Supplementary Material Figure S5.docx]

Supplementary Material


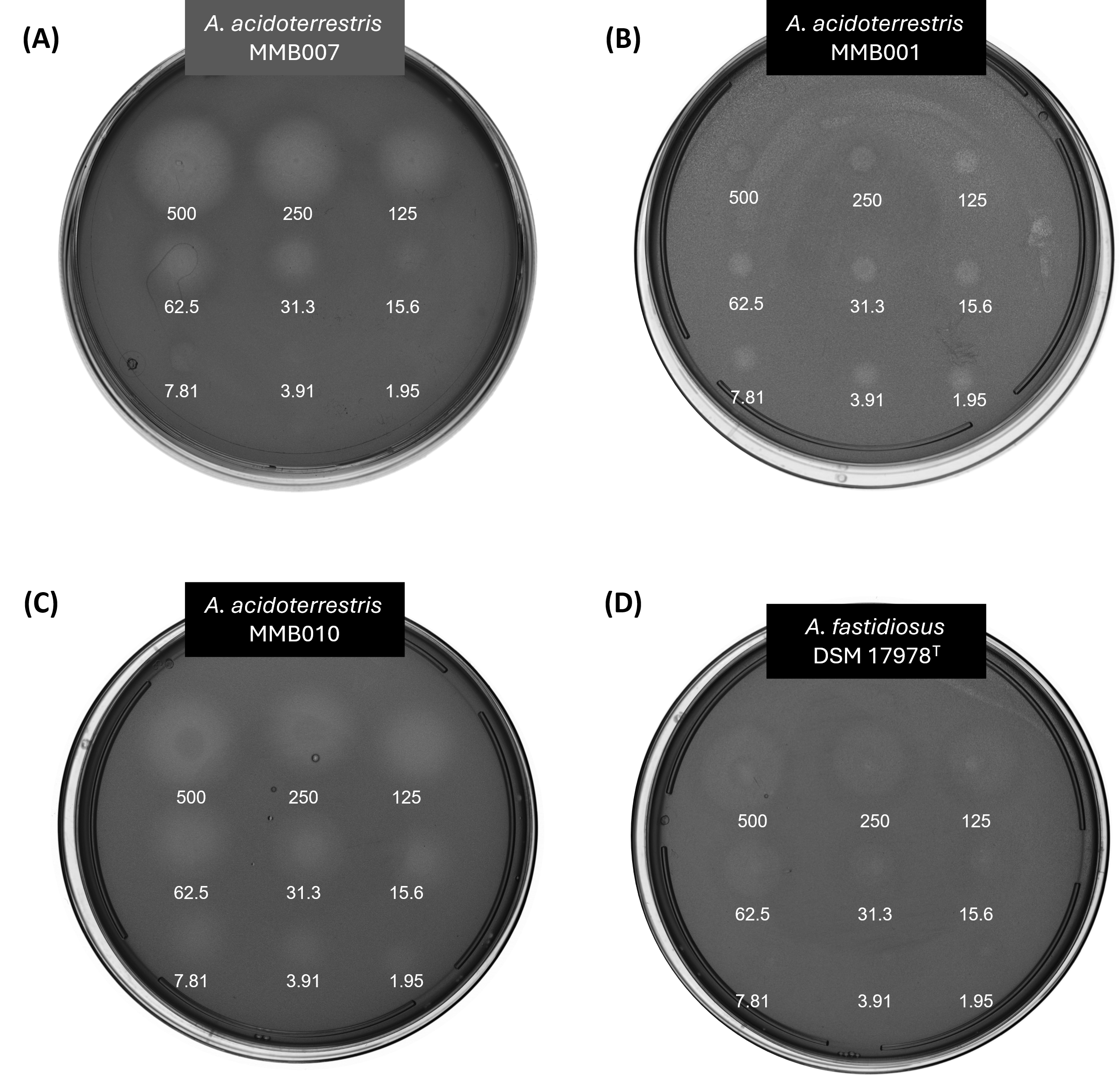


**Supplementary Figure S5.** Inhibitory activity of the recombinant Ali24 against ACB isolates. Serial dilutions of Ali24-His, starting at 500 µg/mL, were spot plated onto lawns of (A) *A acidoterrestris* MMB007, (B) *A. acidoterrestris* MMB001, (C) *A. acidoterrestris* MMB010, and (D) *A. fastidiosus* DSM 17978^T^. Distinct inhibition patterns were observed, with the formation of clearer inhibition halos for *A. acidoterrestris* MMB007 and MMB010, and more turbid halos for the *A. fastidiosus* type strain. A concentration-dependent effect can be observed for all isolates tested except for *A. acidoterrestris* MMB001, which displayed a pattern consistent with a bacteriostatic activity.
