# Supplementary material for: Alicyclobacillin 24: a class III bacteriocin from Alicyclobacillus acidoterrestris targeting species associated with spoilage of acidic fruit-based products
Source: Front Microbiol. 2026 May 1;17:1823210. doi: 10.3389/fmicb.2026.1823210 (PMC13176240; doi:10.3389/fmicb.2026.1823210)
Supplement: Supplementary file 1 [file presentation_1.zip › Supplementary Material Figure S6.docx]

Supplementary Material


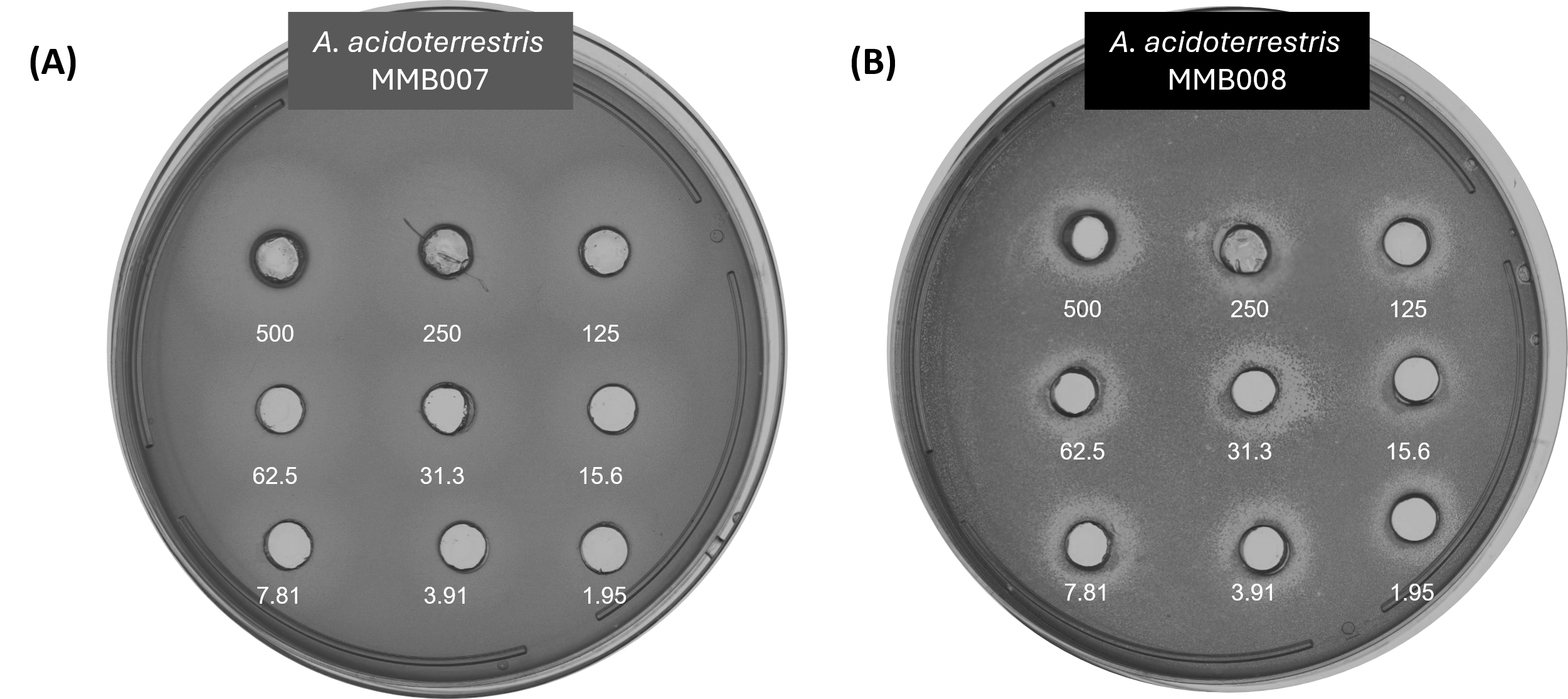


**Supplementary Figure S6.** Agar well diffusion assays assessing the inhibitory activity of Ali24-His against ACB isolates. Serial dilutions of Ali24-His, starting at 500 µg/mL, were tested against (A) *A. acidoterrestris* MMB007, and (B) *A. acidoterrestris* MMB008. Distinct inhibition patterns were observed, with MMB007 producing turbid, concentration-dependent inhibition halos, whereas MMB007 displayed clearer and homogeneous inhibition zones.
