# Supplementary material for: Alicyclobacillin 24: a class III bacteriocin from Alicyclobacillus acidoterrestris targeting species associated with spoilage of acidic fruit-based products
Source: Front Microbiol. 2026 May 1;17:1823210. doi: 10.3389/fmicb.2026.1823210 (PMC13176240; doi:10.3389/fmicb.2026.1823210)
Supplement: Supplementary file 1 [file presentation_1.zip › Supplementary Material Figure S2.docx]

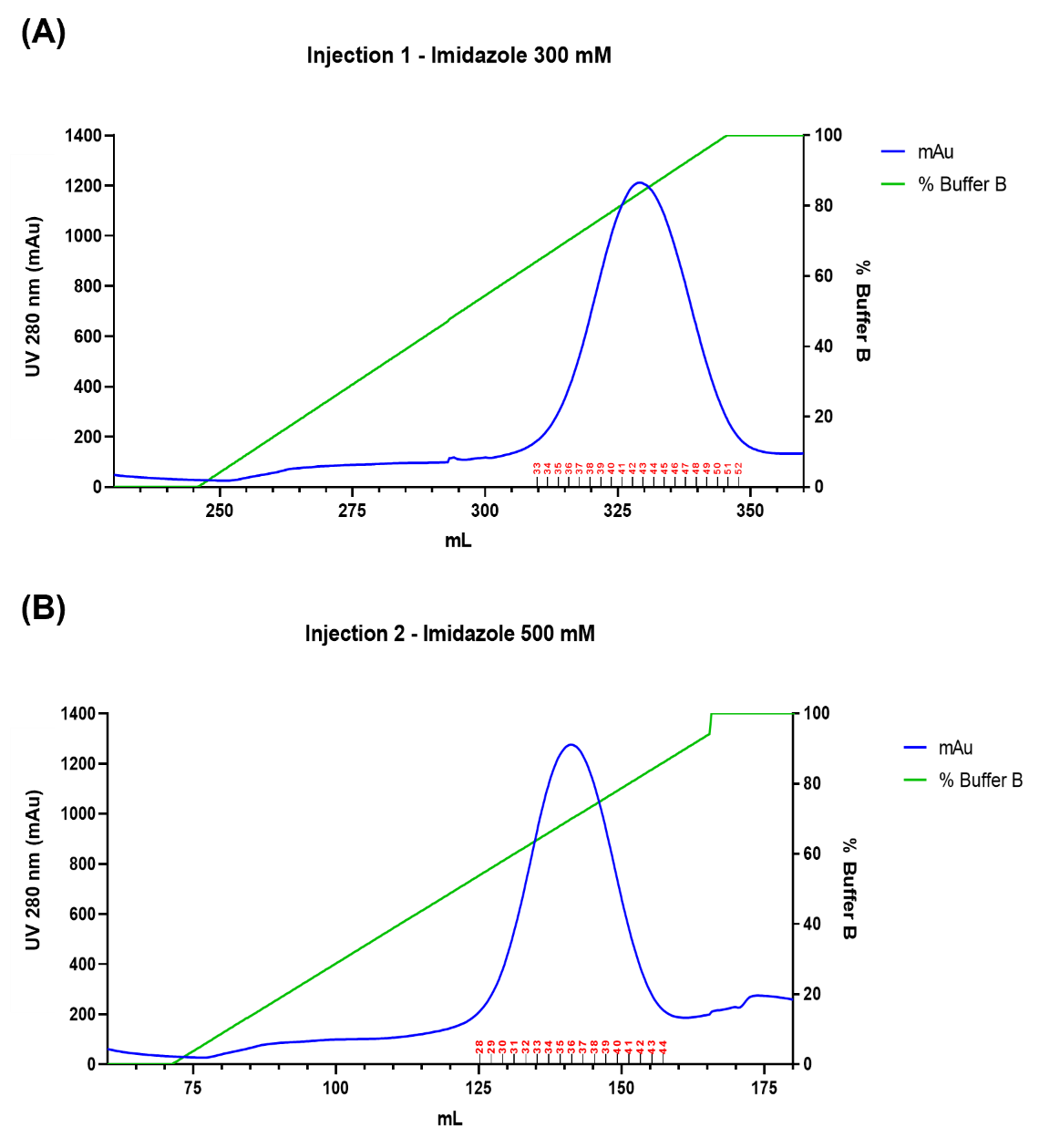


**Figure S2** – Elution gradient of Ali24-His purification using a HisTrap affinity chromatography column. Results are shown for (A) the first injection, eluted with 300 mM imidazole, and (B) the second injection, eluted with 500 mM imidazole. Protein absorbance at 280 nm (mAu) is displayed in blue, the percentage of buffer B (corresponding to increasing imidazole concentration) is displayed in green, and the fractions selected for subsequent analysis are indicated in red.
