# Supplementary material for: Alicyclobacillin 24: a class III bacteriocin from Alicyclobacillus acidoterrestris targeting species associated with spoilage of acidic fruit-based products
Source: Front Microbiol. 2026 May 1;17:1823210. doi: 10.3389/fmicb.2026.1823210 (PMC13176240; doi:10.3389/fmicb.2026.1823210)
Supplement: Supplementary file 2 [file Table_1.docx]

Supplementary Material

**Supplementary Table S1.** List of Protein identifications (FDR<1%) from *A. acidoterrestris* DSM 3922^T^ secretome using LC-MS/MS.

| **#** | **Protein** | **Gene** | **Length** | **Organism** | **Protein Description** | **Coverage** | **Total Peptides** |
| --- | --- | --- | --- | --- | --- | --- | --- |
| 1 | K1I37_00200 |  | 381 |  | K1I37_00200 - hypothetical protein CDS | 19.16 | 4 |
| 2 | K1I37_00995 |  | 516 |  | K1I37_00995 - S8 family serine peptidase CDS | 37.6 | 16 |
| 3 | K1I37_01030 |  | 365 |  | K1I37_01030 - sugar ABC transporter substrate-binding protein CDS | 16.44 | 4 |
| 4 | K1I37_02370 |  | 199 |  | K1I37_02370 - hypothetical protein CDS | 34.17 | 3 |
| 5 | K1I37_02990 |  | 1149 |  | K1I37_02990 - hypothetical protein CDS | 47.52 | 67 |
| 6 | K1I37_03190 |  | 510 |  | K1I37_03190 - peptidoglycan-binding protein CDS | 14.51 | 6 |
| 7 | K1I37_03240 |  | 215 |  | K1I37_03240 - hypothetical protein CDS | 17.21 | 3 |
| 8 | K1I37_03425 |  | 673 |  | K1I37_03425 - peptidoglycan-binding protein CDS | 22.29 | 10 |
| 9 | K1I37_03830 |  | 355 |  | K1I37_03830 - BMP family ABC transporter substrate-binding protein CDS | 31.27 | 7 |
| 10 | K1I37_05905 |  | 344 |  | K1I37_05905 - phosphodiester glycosidase family protein CDS | 18.31 | 4 |
| 11 | K1I37_08310 |  | 576 |  | K1I37_08310 - ABC transporter substrate-binding protein CDS | 53.47 | 27 |
| 12 | K1I37_08685 |  | 340 |  | K1I37_08685 - DUF4309 domain-containing protein CDS | 22.06 | 5 |
| 13 | K1I37_10800 |  | 353 |  | K1I37_10800 - BMP family ABC transporter substrate-binding protein | 60.62 | 14 |
| 14 | K1I37_13065 |  | 572 |  | K1I37_13065 - ABC transporter substrate-binding protein CDS | 41.61 | 28 |
| 15 | K1I37_13150 |  | 381 |  | K1I37_13150 - pstS CDS | 28.87 | 7 |
| 16 | K1I37_15305 |  | 484 |  | K1I37_15305 - hypothetical protein CDS | 10.33 | 3 |
| 17 | K1I37_15405 |  | 368 |  | K1I37_15405 - hypothetical protein CDS | 8.15 | 2 |
| 18 | K1I37_17355 |  | 522 |  | K1I37_17355 - ABC transporter substrate-binding protein CDS | 46.55 | 18 |
| 19 | K1I37_19375 |  | 570 |  | K1I37_19375 - ABC transporter substrate-binding protein CDS | 13.86 | 6 |
| 20 | K1I37_19525 |  | 423 |  | K1I37_19525 - flagellin | 63.83 | 41 |
| 21 | K1I37_19535 |  | 620 |  | K1I37_19535 - flgK CDS | 6.61 | 2 |
| 22 | K1I37_21165 |  | 456 |  | K1I37_21165 - hypothetical protein CDS | 6.14 | 2 |
| **23** | **K1I37_21410** |  | **238** |  | **K1I37_21410 - geobacillin-26 family protein CDS plasmid** | **45.8** | **6** |
| 24 | sp\|P00761\|TRYP_PIG |  | 231 | Sus scrofa | Trypsin | 25.97 | 7 |
| 25 | sp\|P04264\|K2C1_HUMAN | KRT1 | 644 | Homo sapiens | Keratin, type II cytoskeletal 1 | 37.11 | 24 |
| 26 | sp\|P13645\|K1C10_HUMAN | KRT10 | 584 | Homo sapiens | Keratin, type I cytoskeletal 10 | 30.82 | 16 |
| 27 | sp\|P35527\|K1C9_HUMAN | KRT9 | 623 | Homo sapiens | Keratin, type I cytoskeletal 9 | 33.71 | 11 |
| 28 | sp\|P35908\|K22E_HUMAN | KRT2 | 639 | Homo sapiens | Keratin, type II cytoskeletal 2 epidermal | 24.41 | 12 |
| 29 | K1I37_03375 |  | 266 |  | K1I37_03375 - transporter substrate-binding domain-containing protein CDS | 11.28 | 2 |
| 30 | K1I37_19520 |  | 923 |  | K1I37_19520 - fliD CDS | 2.93 | 2 |
| 31 | K1I37_03245 |  | 508 |  | K1I37_03245 - peptidoglycan-binding protein CDS | 9.06 | 4 |
| 32 | K1I37_10110 |  | 284 |  | K1I37_10110 - flagellar hook-basal body complex protein CDS | 13.03 | 1 |
| 33 | K1I37_15340 |  | 496 |  | K1I37_15340 - hypothetical protein CDS | 3.83 | 1 |
| 34 | K1I37_15415 |  | 325 |  | K1I37_15415 - hypothetical protein CDS | 4.31 | 1 |
| 35 | sp\|O77727\|K1C15_SHEEP | KRT15 | 453 | Ovis aries | Keratin, type I cytoskeletal 15 | 8.61 | 4 |
| 36 | K1I37_17130 |  | 141 |  | K1I37_17130 - glycosyl hydrolase family 18 protein CDS | 9.93 | 1 |
| 37 | K1I37_03730 |  | 527 |  | K1I37_03730 - DUF4855 domain-containing protein CDS | 2.66 | 1 |
| 38 | K1I37_09415 |  | 287 |  | K1I37_09415 - MetQ/NlpA family ABC transporter substrate-binding protein CDS | 4.18 | 1 |
| 39 | K1I37_13000 |  | 1073 |  | K1I37_13000 - adenosylcobalamin-dependent ribonucleoside-diphosphate reductase CDS | 0.75 | 1 |
| 40 | K1I37_07640 |  | 395 |  | K1I37_07640 - hypothetical protein CDS | 2.78 | 1 |
| 41 | K1I37_00920 |  | 502 |  | K1I37_00920 - MFS transporter CDS | 5.18 | 1 |
| 42 | K1I37_15410 |  | 133 |  | K1I37_15410 - hypothetical protein CDS | 8.27 | 1 |
| 43 | K1I37_03690 |  | 366 |  | K1I37_03690 - pilM CDS | 2.19 | 1 |
| 44 | K1I37_12840 |  | 179 |  | K1I37_12840 - small multi-drug export protein CDS | 4.47 | 1 |
| 45 | K1I37_00420 |  | 992 |  | K1I37_00420 - DEAD/DEAH box helicase family protein CDS | 0.81 | 1 |
| 46 | K1I37_17655 |  | 107 |  | K1I37_17655 - cupin domain-containing protein CDS | 26.17 | 1 |
| 47 | K1I37_20015 |  | 157 |  | K1I37_20015 - flavin reductase family protein CDS | 6.37 | 1 |
| 48 | K1I37_19400 |  | 221 |  | K1I37_19400 - wrbA CDS | 19 | 1 |
